# Short interpregnancy interval can lead to adverse pregnancy outcomes: A meta-analysis

**DOI:** 10.3389/fmed.2022.922053

**Published:** 2022-11-30

**Authors:** Yumei Wang, Can Zeng, Yuhong Chen, Liu Yang, Di Tian, Xinghui Liu, Yonghong Lin

**Affiliations:** ^1^Department of Health Care, Chengdu Women’s and Children’s Central Hospital, School of Medicine, University of Electronic Science and Technology of China, Chengdu, China; ^2^Department of Obstetrics and Gynecology, West China Second University Hospital, Sichuan University, Chengdu, China; ^3^Key Laboratory of Birth Defects and Related Diseases of Women and Children, Ministry of Education, West China Second University Hospital, Sichuan University, Chengdu, China; ^4^Department of Travel to Check, Customs of Chengdu Shuangliu Airport Belongs to Chengdu Customs, Chengdu, China

**Keywords:** interpregnancy interval, adverse pregnancy outcomes, preterm birth, gestational hypertension, gestational diabetes

## Abstract

**Background:**

The evidence of some previous papers was insufficient in studying the causal association between interpregnancy interval (IPI) and adverse pregnancy outcomes. In addition, more literature have been updated worldwide during the last 10 years.

**Methods:**

English and Chinese articles published from January 1980 to August 2021 in the databases of PubMed, Cochrane Library, Ovid, Embase, China Biology Medicine disc (CBM), and China National Knowledge Infrastructure (CNKI) were searched. Then following the inclusion and exclusion criteria, we screened the articles. Utilizing the Newcastle–Ottawa Scale (NOS), we evaluated the quality of the included articles. The literature information extraction table was set up in Excel, and the meta-analysis was performed with Stata 16.0 software (Texas, USA).

**Results:**

A total of 41 articles were included in the meta-analysis, and NOS scores were four to eight. The short IPI after delivery was the risk factor of preterm birth (pooled odds ratio 1.49, 95% confidence interval 1.42–1.57), very preterm birth (pooled OR: 1.82, 95% CI: 1.55–2.14), low birth weight (pooled OR: 1.33, 95% CI: 1.24–1.43), and small for gestational age (pooled OR: 1.14, 95% CI: 1.07–1.21), offspring death (pooled OR: 1.60, 95% CI: 1.51–1.69), NICU (pooled OR: 1.26, 95% CI: 1.01–1.57), and congenital abnormality (pooled OR: 1.10, 95% CI: 1.05–1.16), while was not the risk factor of gestational hypertension (pooled OR: 0.95, 95% CI: 0.93–0.98) or gestational diabetes (pooled OR: 1.06, 95% CI: 0.93–1.20).

**Conclusion:**

Short IPI (IPI < 6 months) can lead to adverse perinatal outcomes, while it is not a risk factor for gestational diabetes and gestational hypertension. Therefore, more high-quality studies covering more comprehensive indicators of maternal and perinatal pregnancy outcomes are needed to ameliorate the pregnancy policy for women of childbearing age.

## Introduction

Interpregnancy interval (IPI) is the period between the previous delivery and the following pregnancy. Previous studies have found that the length of IPI was related to adverse perinatal outcomes, such as preterm birth, low birth weight, small for gestational age, and stillbirth (1). In particular, 18–23 months IPI could promote maternal and fetal outcomes (2–4), while short IPI (IPI less than 6 months) was significantly associated with an increased risk of preterm birth, low birth weight, and small for gestational age (1). Therefore, WHO recommended that the IPI after delivery should be more than 24 months (5).

Previous meta-analyses have explored the relationship between short IPI and adverse pregnancy outcomes (6, 7). However, the included literature varies in its quality, for example, the analyzed studies were cross-sectional studies, and the birth interval (the time interval between two live births) rather than the IPI was measured. Thus, the evidence of meta-analysis was insufficient in studying the causal association between IPI and adverse pregnancy outcomes (8). A systematic review (7) found that short IPIs in high-resource settings may be associated with an increased risk of maternal obesity and gestational diabetes, as well as a reduced risk of preeclampsia in the next pregnancy. However, most pregnancy outcomes from the systematic review were evaluated in a single study, and the supportive evidence of associations is insufficient, and there were few studies focused on the influence of short IPIs on maternal morbidity and mortality.

The most recent meta-analysis on the effects of short gestation intervals on fetal and maternal outcomes was published in 2012 (6), which was 10 years old and lacked research from some countries, such as China. Previous systematic reviews and meta-analyses have examined the influence of short IPIs on perinatal mortality outcomes, but the researchers compared the IPI < 6 months with IPI ≥ 6 months and calculated the combined effect values, so these researches could limit the results (9). Through meta-analysis, this study discussed the influence of short IPI on adverse pregnancy outcomes and provided a basis and guidance for women of childbearing age to choose the appropriate IPI. Choosing an appropriate IPI is vital to protect mothers and babies, perfect the quality of birth population, and reduce the occurrence of birth defects.

## Materials and methods

### Search methods

English and Chinese articles were searched in the databases of PubMed, Cochrane Library, Ovid, Embase, China Biology Medicine disc (CBM), and China National Knowledge Infrastructure (CNKI). The search formula was: (“birth intervals” [Mesh] OR “interpregnancy interval” [Text Word] OR “birth interval” [Text Word] OR “interbirth interval” [Text Word] OR “pregnancy spacing” [Text Word] OR “pregnancy interval” [Text Word] OR “birth spacing” [Text Word]) AND (“pregnancy outcome” [Mesh] OR “infant, low birth weight” [Mesh] OR “premature birth” [Mesh] OR “infant, small for gestational age” [Mesh] OR “fetal growth retardation” [Mesh] OR “intensive care units, neonatal” [Mesh] OR “fetal death” [Mesh] OR “stillbirth” [Mesh] OR “perinatal death” [Mesh] OR “fetal mortality” [Mesh] OR “perinatal mortality” [Mesh] OR “infant mortality” [Mesh] OR “congenital abnormalities” [Mesh] OR “diabetes, gestational” [Mesh] OR “hypertension, pregnancy-induced” [Mesh] OR “pre-eclampsia” [Mesh] OR “hypertensive disorders” [Text Word] OR “maternal morbidity” [Text Word] OR “maternal mortality” [Mesh] OR “maternal death” [Mesh] OR “uterine rupture” [Mesh] OR “abruptio placentae” [Mesh] OR “placenta previa” [Mesh] OR “obesity” [Mesh] OR “dystocia” [Mesh]). The search ranged from Chinese articles to English articles published from January 1980 to August 2021.

### Criteria for inclusion and exclusion of literature

The inclusion and exclusion criteria were determined according to PECO (Population, Exposure, Control and Outcome).

#### Inclusion criteria

1) Cohort study and case–control study-based population.

2) At least one birth and second pregnancy.

3) The definition of IPI was the interval from the last delivery to the beginning of the next pregnancy (the last menstrual date).

4) Defined short IPI (IPI < 6 months) and reference IPI. Previous studies have found a J-shaped relationship between IPI and adverse pregnancy outcomes (2, 4). Therefore, upper and lower limits must be clearly defined with reference IPI to reduce bias caused by the misclassification of IPI.

5) At least one pregnancy outcome analyzed.

6) IPI, a grouping variable, the OR, RR, and 95% confidence interval (CI) of association between different IPIs and pregnancy outcomes were reported.

#### Exclusion criteria

1) Experimental study and cross-sectional study.

2) Analyzed birth interval without IPI.

3) IPI was a quantitative continuous variable.

4) Summary, abstract.

5) OR, RR, and 95% CI were not reported, or the statistics above cannot be calculated according to the original data.

6) Excluded the studies only about inter-pregnancy interval after unnatural pregnancy, preterm birth, stillbirth, and pregnancy loss or termination. Considering the use of assisted reproductive technology, and the adverse outcomes of the previous pregnancy, such as preterm birth, stillbirth, and termination of pregnancy, may be caused by maternal health status, genetic-related and other factors, which may also affect the subsequent pregnancy interval and the pregnancy outcomes. So, in those studies, there was potential for confounding bias, and the external validity was low.

### Literature screening

The first and second authors read and screened the titles and abstracts of the retrieved articles and excluded irrelevant articles by following the inclusion criteria and exclusion criteria preliminarily.

### Literature evaluation

The first and second authors evaluated the contained studies independently, using Newcastle–Ottawa Scale (NOS) (10) and excluded the studies with NOS score ≤ 4. The main studies included in the assessment were selection of subjects, measurement of exposure factors, inter-group comparability, and follow-up. For studies with inconsistent evaluation results by two screeners, judgment was made through mutual consultation.

### Statistical analysis

#### Data extraction

Excel was used to establish the extraction table for literature information; extraction contents include study implementers, study site, study subjects, sample size, observation period, exposure measurements, outcome indicators, study results, and controlled confounding factors. When combining effect values, we used the method of Hamling et al. (11) to convert OR value or RR value, because different reference groups may be selected in multivariate analysis of different studies. Stata 16.0 software (Texas, USA) was applied to analyze the included studies statistically.

#### Heterogeneity test

The heterogeneity of the study was tested by *I*^2^ test. If *P* > 0.05 and *I*^2^ < 50%, it meant that the study was homogeneous and applied the fixed effects model; If *P* < 0.05, or *I*^2^ > 50%, it meant that the study was heterogeneous, and further subgroup analysis was made by factors such as study area, study type, and the outcome of the previous pregnancy. If the heterogeneity still existed after removing the study that had a great influence on the merger effect, the random-effects model was adopted.

#### Sensitivity analysis

Three sensitivity analyses were performed. First, after each study was removed from the meta-analysis of pregnancy outcomes, the combined effect value was recalculated. Second, the combined effect value was recalculated after the references with NOS score ≤ 4 were excluded. Third, for meta-analyses with no more than four references included, both random-effects model and fixed effects model were used. Before and after sensitivity analysis, if the confidence interval of the combined statistics changes from *P* > 0.05 to *P* < 0.05 or from *P* < 0.05 to *P* > 0.05, or the variation range of the combined OR value exceeds 10%, it indicates that the deleted references are outliers with important influence.

#### Publication bias

Egger’s test was used to evaluate publication bias. If *P* > 0.05, the risk of publication bias was considered to be low.

#### Assessment of evidence quality

The GRADE (The Grades of Recommendation, Assessment, Development, and Evaluation) (12) system was used to evaluate the quality of evidence. The risk of bias, inconsistencies, inaccuracies, indirection, and the bias of reporting were investigated.

## Results

### Literature characteristics

**FIGURE 1:**
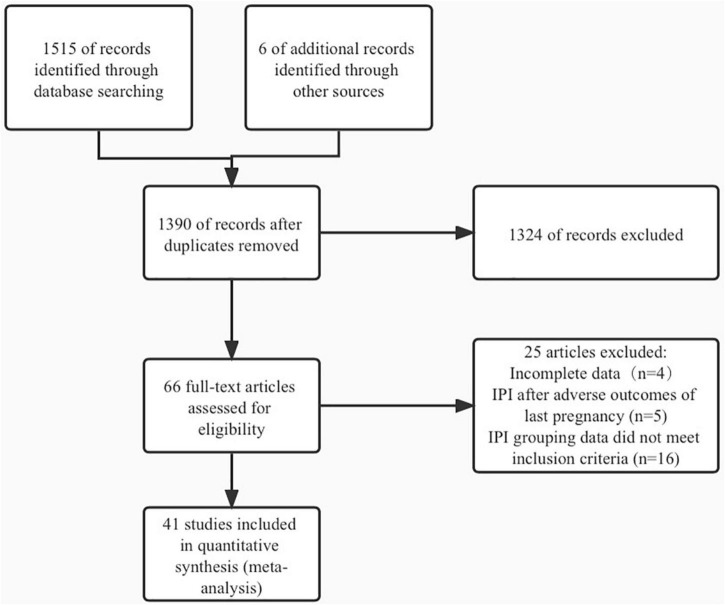
Literature screening process.

Through searching the database, 1,499 English articles and 16 Chinese articles were searched, and six articles were supplemented by systematic review and meta-analysis. After the exclusion of duplicates, 1,390 articles remained. After subsequently reading the title and abstract, the researchers excluded 1,324 articles. Finally, a total of 41 articles (1–3, 13–50) were included (Supplementary Table 2) in the meta-analysis after further reading the full text. Supplementary Table 1 showed the characteristics of the excluded studies. Among the 25 articles excluded, four were excluded because of incomplete data, five for the IPI after adverse outcomes of the last pregnancy, and 16 for IPI grouping data did not meet inclusion criteria. Literature screening process is shown in Figure 1. Most of the studies included in the meta-analysis were in European and American countries: the United States (22), Canada (6), United Kingdom (1), the Netherlands (3), Sweden (1), Israel (1), China (1), Australia (4), and multi-countries (2). The NOS scores of the 41 articles included in the meta-analysis ranged from four to eight, and the characteristics of the articles are shown in Supplementary Table 2.

When analyzing the association between different IPIs and pregnancy outcomes, three studies (14, 25, 38) did not use 18–23 months as the reference group. Therefore, the data of the reference group were transformed when the effect values were combined.

Among the included studies, the Lieberman et al. (13) study and the Lang et al. (14) study were the same population with different outcomes. The Schummers et al. (37) study and the Schummers et al. (47) study were the same database and population. The Tessema et al. (48) study and the Marinovich et al. (49) study were the same database and population. The vast majority of studies were based on population-based birth registration information systems. In the analysis of the association between IPI and pregnancy outcome, the following covariables were included in the studies: maternal age (39 articles); mother’s sociodemographic characteristics, such as race/ethnicity (29 articles), educational level (23 articles), marital status (18 articles), and economic status (12 articles), smoking before or during pregnancy (26 articles), drinking alcohol before or during pregnancy (10 articles), parity (17 articles), and prenatal care (11 articles). Sixteen studies controlled the outcome of a previous pregnancy, such as preterm birth.

Based on the inclusion and exclusion criteria, nine pregnancy outcomes were included in the meta-analysis, including preterm birth, very preterm birth, low birth weight, small for gestational age infants, offspring death, neonatal intensive care (NICU), congenital abnormality, gestational hypertension, and gestational diabetes. In light of pregnancy outcomes, the heterogeneity test and publication bias test of the studies included in the meta-analysis were carried out, as shown in Table 1. The result of Egger’s test of each outcome index was *P* > 0.05, which suggested that the publication bias of studies included in the meta-analysis was small.

**TABLE 1:** Heterogeneity test and publication bias test of the meta-analysis of various pregnancy outcomes.

		Heterogeneity test		Egger’s test			
Maternal and infant outcome	Number of articles	*I* ^2^	*P* of Cochran’s *Q*-test	*t*	*P*	Effect model	Quality evidence
Preterm birth	28	92.0%	<0.001	–1.77	0.088	Random-effects model	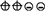
Very preterm birth	13	89.0%	<0.001	0.74	0.476	Random-effects model	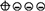
Low birth weight	18	84.7%	<0.001	–1.72	0.105	Random-effects model	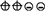
Small for gestational age infants	23	83.8%	<0.001	–0.88	0.386	Random-effects model	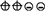
Offspring death	6	0	0.719	0.60	0.583	Fixed effects model	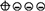
NICU	4	90.3%	<0.001	–0.97	0.511	Random-effects model	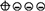
Congenital abnormality	4	0	0.458	–0.47	0.683	Fixed effects model	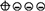
Gestational hypertension	6	2.7%	0.399	–0.49	0.652	Fixed effects model	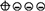
Gestational diabetes	6	92.8%	<0.001	2.64	0.058	Random-effects model	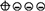

### The influence of short interpregnancy interval on the adverse outcomes of perinatal infants

#### The influence of short interpregnancy interval on preterm birth

**FIGURE 2:**
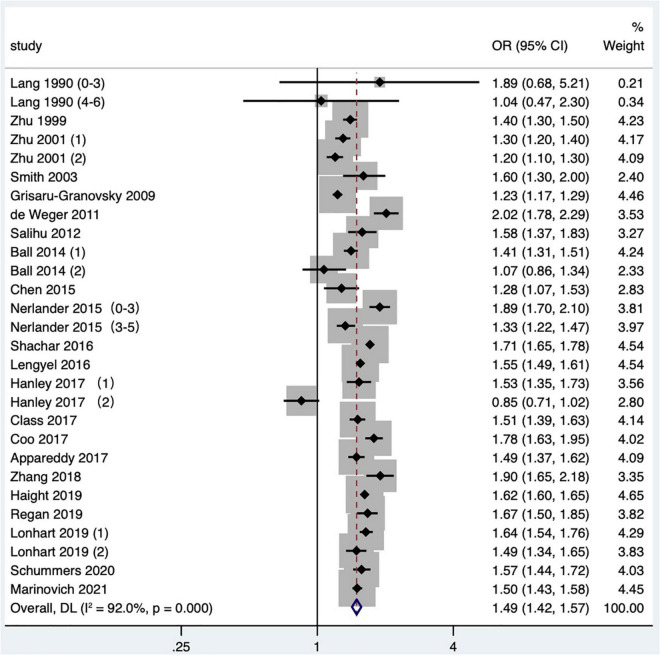
Meta-analysis forest map of the effect of short IPI on preterm birth. Lang et al. (14) (0–3) referred to the IPI of 0–3 months vs. 18–23 months; Lang et al. (14) (4–6) referred to the IPI of 4–6 months vs. 18–23 months; Nerlander et al. (27) (0–3) referred to the IPI of 0–3 months vs. 18–23 months; Nerlander et al. (27) (3–5) referred to the IPI of 3–5 months vs. 18–23 months; Zhu et al. (15) (1) was white data, Zhu et al. (15) (2) was black data; Hanley et al. (36) (1) was non-matching data, and Hanley et al. (36) (2) was within-mother matching data; Ball et al. (24) (1) was non-matching data, Ball et al. (24) (2) was within-mother matching data; Lonhart et al. (43) (1) was non-Hispanic whites data, and Lonhart et al. (43) (2) was non-Hispanic blacks data.

A total of 23 studies (2, 14–16, 19–21, 24, 25, 27, 29–31, 34–36, 38, 41–43, 47–49) met the inclusion criteria, most of which were in North America, two of which covered four countries: the United States, Australia, Finland, and Norway (48, 49), two were in Asia, three were in Europe, and two were in Australia. Although the Tessema et al. (48) study and the Marinovich et al. (49) study were derived from the same database at the same time period, considering that the Marinovich et al. (49) study focused on the effect of pregnancy interval after full-term pregnancy on preterm birth; therefore, the Marinovich et al. (49) study was preferentially selected for the meta-analysis. At last, 22 articles (2, 14–16, 19–21, 24, 25, 27, 29–31, 34–36, 38, 41–43, 47, 49) with 28 research data were selected for meta-analysis. The definition of preterm birth was that the gestational age at delivery was less than 37 weeks, but one article (16) defined preterm birth as 33–36 weeks. The results showed that short IPI (IPI < 6 months) after delivery was a risk factor for preterm birth (pooled OR: 1.49, 95% CI: 1.42–1.57; Figure 2). After each study was removed seriatim from the sensitivity analysis, the pooled OR values varied from 1.48 to 1.52, the minimum 95% CI lower limit of 1.40, and the maximum 95% CI upper limit of 1.59. After removing the matched data (the data about with-in mother comparisons) of the Hanley et al. (36) study and the Ball et al. (24) study, the pooled OR changed a little (1.53, 95% CI: 1.46–1.60). Similarly, we removed the study with NOS score of 4 (15) (pooled OR: 1.52, 95% CI: 1.45–1.59). When we removed the study with women under 20 years old (27), it didn’t change the relationship between short IPI and preterm birth (pooled OR: 1.49, 95% CI: 1.41–1.56; *I*^2^ = 92.0%, *P* < 0.001).

#### The influence of short interpregnancy interval on very preterm birth

**FIGURE 3:**
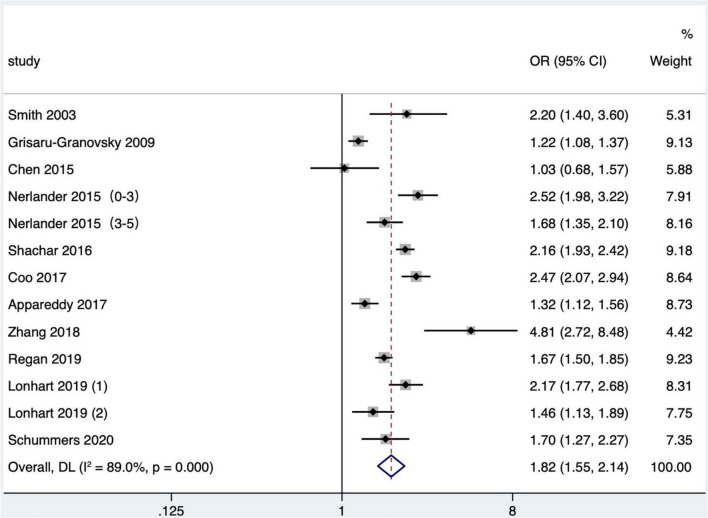
Meta-analysis forest map of the effect of short IPI on very preterm birth. Nerlander et al. (27) (0–3) referred to the IPI of 0–3 months vs. 18–23 months; Nerlander et al. (27) (3–5) referred to the IPI of 3–5 months vs. 18–23 months; Lonhart et al. (43) (1) was non-Hispanic whites data, and Lonhart et al. (43) (2) was non-Hispanic blacks data.

Eleven articles (16, 19, 25, 27, 29, 31, 35, 38, 42, 43, 47) (13 research data) classified preterm birth according to gestational age at delivery. When analyzing very preterm birth, a meta-analysis found that four studies only provided the upper limit of very preterm birth, including one article that defined very preterm birth as less than 32 weeks (27), one less than 33 weeks (19), and two less than 34 weeks (31, 35). Five studies defined the range of very preterm birth as 28–34 weeks of gestational age at delivery (25), 24–31 weeks (29), 24–32 weeks (16), 26–32 weeks (38), and 28–32 weeks (42), respectively. One study (43) did not define very preterm birth but reported a subgroup analysis of preterm births between 24 and 31 gestational weeks, and one (47) studied the preterm birth before 28 gestational weeks, both of which were included in the meta-analysis. Meta-analysis exhibited that short IPI (IPI < 6 months) was the risk factor for very preterm birth (pooled OR: 1.82, 95% CI: 1.55–2.14; Figure 3). After removing each study seriatim, the pooled OR value varied from 1.74 to 1.89, with the minimum 95% CI lower limit of 1.49 and the maximum 95% CI upper limit of 2.23. After removing the study of Nerlander et al. (27), the relationship between short IPI and very preterm birth didn’t change (pooled OR: 1.78, 95% CI: 1.49–2.14; *I*^2^ = 90.0%, *P* < 0.001).

#### The influence of short interpregnancy interval on low birth weight

**FIGURE 4:**
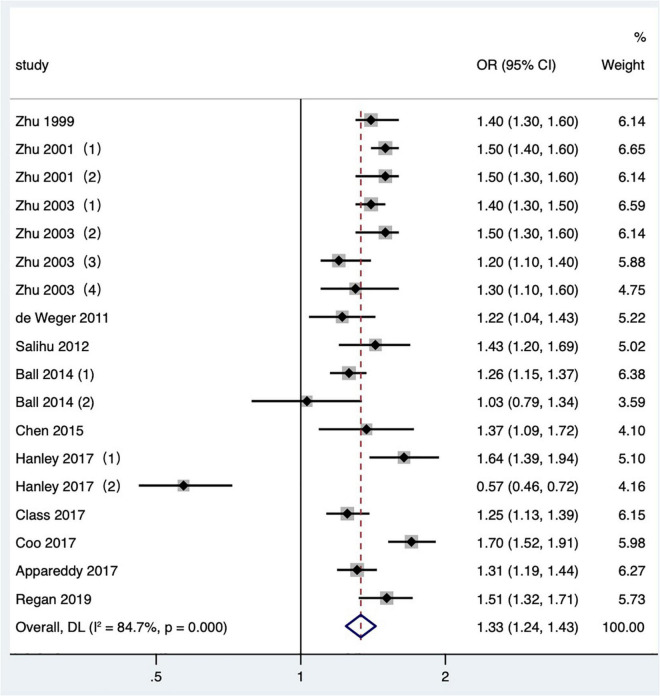
Meta-analysis forest map of the effect of short IPI on low birth weight. Zhu et al. (15) (1) was white data, Zhu et al. (15) (2) was black data; Zhu and Le (3) (1) studied the IPI between the first pregnancy and the second pregnancy; Zhu and Le (3) (2) studied the IPI between the second and third pregnancy; Zhu and Le (3) (3) studied the IPI between the third and fourth pregnancy; Zhu and Le (3) (4) studied the IPI between the fourth and fifth pregnancy; Ball et al. (24) (1) was non-matching data, Ball et al. (24) (2) was within-mother matching data; Hanley et al. (36) (1) was non-matching data, and Hanley et al. (36) (2) was within-mother matching data.

Twelve articles (2, 3, 15, 20, 21, 24, 25, 31, 34–36, 42) with 18 research data were included in the meta-analysis. Low birth weight is defined as birthweight < 2,500 g. The data reported by Zhu and Le (3) were divided into four groups of IPI according to parity. Hanley et al. (36) and Ball et al. (24) reported the comparison of non-matching among mothers and the comparison of matching between two consecutive IPIs of the same mother, respectively. The results showed that the short IPI after delivery (IPI < 6 months) was the risk factor for low birth weight (pooled OR: 1.33, 95% CI: 1.24–1.43; Figure 4). After removing each study seriatim, the pooled OR value varied from 1.31 to 1.38, with the minimum 95% CI lower limit of 1.22 and the maximum 95% CI upper limit of 1.46. After removing the matched data in two studies (24, 36), the odds ratio changed a little (pooled OR: 1.40, 95% CI: 1.33–1.47). In the study of Zhu and Le (3), removing the IPI after the second birth had little effect on the odds ratio (pooled OR: 1.33, 95% CI: 1.23–1.45; *I*^2^ = 86.4%, *P* < 0.001), which was the same as removing the study with NOS score of 4 (15) (pooled OR: 1.31, 95% CI: 1.21–1.42; *I*^2^ = 85.1%, *P* < 0.001).

#### The effect of short interpregnancy interval on small-for-gestational-age

**FIGURE 5:**
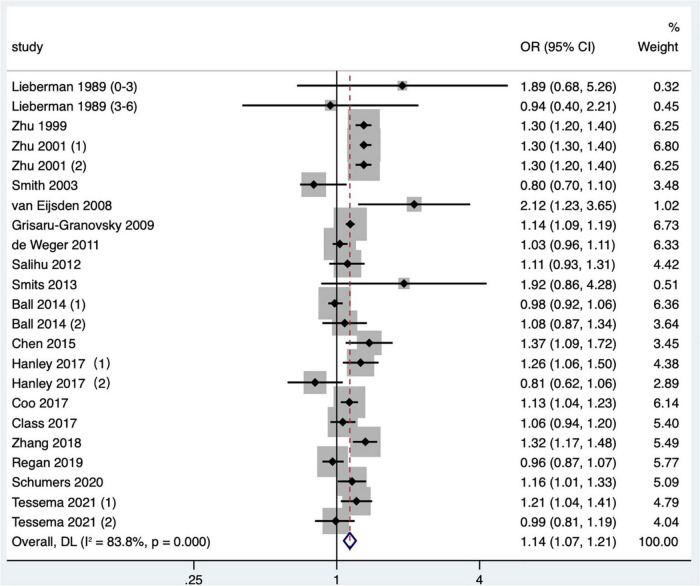
Meta-analysis forest map of the effects of short IPI on small for gestational age infants. Lieberman et al. (13) (0–3) referred to the IPI of 0–3 months vs. 18–23 months; Lieberman et al. (13) (3–6) referred to the IPI of 3–6 months vs. 18–23 months; Zhu et al. (15) (1) was white data, Zhu et al. (15) (2) was black data; Ball et al. (24) (1) was non-matching data, and Ball et al. (24) (2) was within-mother matching data; Hanley et al. (36) (1) was non-matching data, and Hanley et al. (36) (2) was intra-maternal matching data; Tessema et al. (48) (1) was non-matching data, and Tessema et al. (48) (2) was within-mother matching data.

Eighteen articles (2, 13, 15, 16, 18–21, 23–26, 34–36, 38, 42, 47, 48) (23 research data) were contained in the meta-analysis. Data from the Schummers et al. (37) study were not reported in the main findings, so only the data from the Schummers et al. (47) study were included in the quantitative synthesis. For the definition of small for gestational age, eight articles (2, 24, 35, 36, 38, 42, 47, 48) were birthweight less than 10th percentile of sex and gestational age-specific birthweight based on given standards; three articles (18, 20, 23) were birthweight less than 10th percentile of a given sex, parity, and gestational age; one article (15) was birthweight less than 10th percentile of a given gestational age, race, sex, and parity; one article (34) was that the birthweight was more than two standard deviations below the average weight of a given gestational age, and one article (16) was birthweight less than fifth percentile among the birthweight of live births; four articles (13, 19, 21, 25) were birthweight less than 10th percentile of a given gestational age. The result showed that the short IPI (IPI < 6 months) was a risk factor for small for gestational age infants (pooled OR: 1.14, 95% CI: 1.07–1.21; Figure 5). After removing each study seriatim, the pooled OR value varied from 1.12 to 1.15, with the minimum 95% CI lower limit of 1.06 and the maximum 95% CI upper limit of 1.22. Removing the matching data (24, 36, 48) had little effect on the odds ratio (pooled OR: 1.16, 95% CI: 1.09–1.23), which was the same as removing the study with NOS score of 4 (15) (pooled OR: 1.11, 95% CI: 1.05–1.18; *I*^2^ = 75.1%, *P* < 0.001).

#### The effect of short interpregnancy interval on offspring death

**FIGURE 6:**
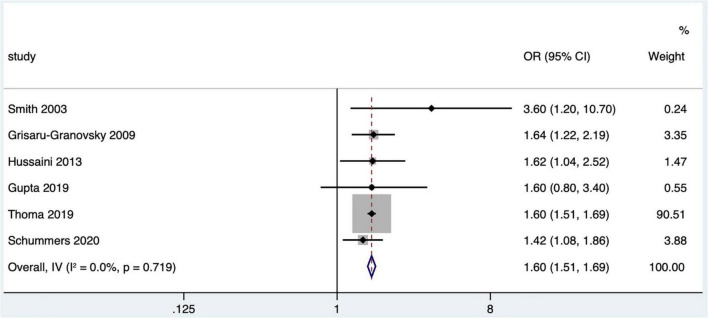
Meta-analysis forest map of the influence of short IPI on offspring death.

Nine studies (16, 19, 22, 25, 31, 32, 40, 44, 47) met the literature screening criteria, and the outcome indicators of fetal/infant death included stillbirth (fetal death at gestational age ≥ 20 weeks) (40), perinatal death (16, 25, 47), neonatal death (19, 22, 44), and infant death (22, 31, 32, 44). Schummers et al. (47) studied perinatal mortality including stillbirth and neonatal death within 28 days after birth. Data on the relationship between IPI and offspring death in one study (25) were not available and not included in the quantitative combination. As the cause of infant death within 1-year-old may be affected by a variety of confounding factors, so the study data with infant death as the outcome indicator were not included in the quantitative combination. Finally, six (16, 19, 22, 40, 44, 47) studies were included in the meta-analysis, including two case–control studies (22, 40) and four cohort studies (16, 19, 44, 47). Meta-analysis with a fixed effects model showed that short IPI (IPI < 6 months) was a risk factor for fetal/infant death (pooled OR: 1.60, 95% CI: 1.51–1.69; Figure 6). After removing each study one by one, the pooled OR values ranged from 1.57 to 1.60, with the minimum 95% CI lower limit of 1.32 and the maximum 95% CI upper limit of 1.87.

#### The influence of short interpregnancy interval on neonatal intensive care

**FIGURE 7:**
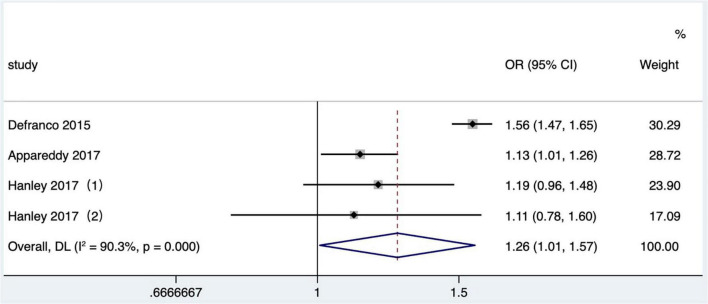
Meta-analysis forest map of the influence of short IPI on NICU. Hanley et al. (36) (1) was non-matching data, and Hanley et al. (36) (2) was within-mother matching data.

Four studies (25, 26, 31, 36) met the literature inclusion criteria, all of which were published after 2010 in North America, and the outcome indicator was NICU admission. Data on the NICU admission in one study (25) were not available and not included in the quantitative combination. One study (26) only reported the number of NICU admission cases in different IPI groups, so we combined the effect values after calculating crude OR values according to the number. One study (36) reported the comparison between mothers and the comparison of matching data between two consecutive IPIs of the same mother, respectively. At last, three articles (26, 31, 36) with four research data were selected in our meta-analysis. Short IPI (IPI < 6 months) was the risk factor for NICU (pooled OR: 1.26, 95% CI: 1.01–1.57; Figure 7). After removing each study seriatim, the pooled OR values varied from 1.14 to 1.32, with the minimum 95% CI lower limit of 0.97 and the maximum 95% CI upper limit of 1.68. After removing the matching data of Hanley et al. (36), the pooled OR value changed little (pooled OR: 1.29, 95% CI: 1.01–1.65), but short IPI was no longer being a risk factor for NICU after removing the non-matching data (pooled OR: 1.28, 95% CI: 0.97–1.67).

#### Influence of short interpregnancy interval on congenital abnormality

**FIGURE 8:**
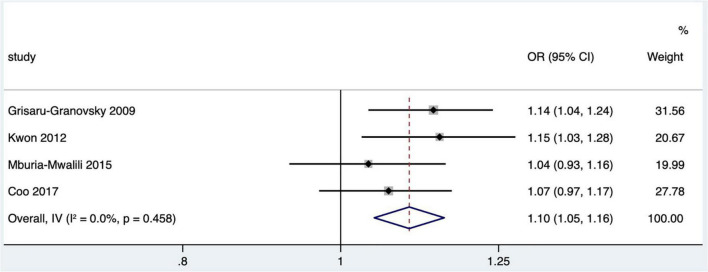
Meta-analysis forest map of the influence of short IPI on congenital abnormality.

Four studies (1, 19, 28, 33) met the literature inclusion criteria. The outcome indicator was the congenital abnormality of offspring. In one article, the outcome indicator was major congenital malformation (19), one was congenital abnormality (33) and two were birth defects (1, 28), and the diagnostic criteria were based on ICD-9-CM 740 to 759 or ICD-10-CA Q00-Q99. Short IPI was a risk factor for congenital abnormality (pooled OR: 1.10, 95% CI: 1.05–1.16; Figure 8). After removing each study seriatim, the pooled OR values varied from 1.08 to 1.12, with the minimum 95% CI lower limit of 1.02 and the maximum 95% CI upper limit of 1.18.

#### Subgroup analysis of adverse perinatal outcomes caused by short interpregnancy interval

**FIGURE 9:**
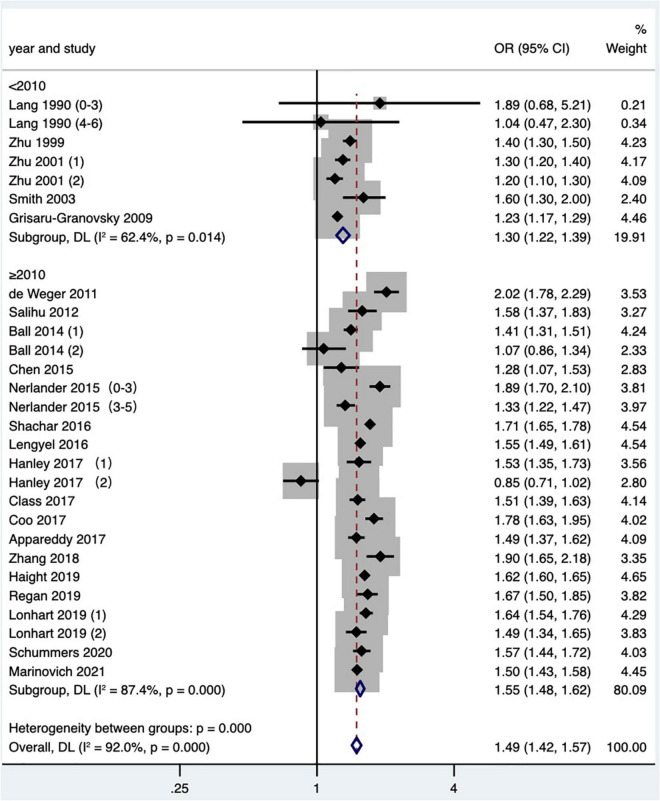
Subgroup analysis of the effect of short IPI on preterm birth. Lang et al. (14) (0–3) referred to the IPI of 0–3 months vs. 18–23 months; Lang et al. (14) (4–6) referred to the IPI of 4–6 months vs. 18–23 months; Zhu et al. (15) (1) was white data, Zhu et al. (15) (2) was black data; Nerlander et al. (27) (0–3) referred to the IPI of 0–3 months; Nerlander et al. (27) (3–5) referred to the IPI of 3–5 months; Ball et al. (24) (1) was non-matching data, and Ball et al. (24) (2) was within-mother matching data; Hanley et al. (36) (1) was non-matching data, and Hanley et al. (36) (2) was within-mother matching data; Lonhart et al. (43) (1) was non-Hispanic whites data, and Lonhart et al. (43) (2) was non-Hispanic blacks data.

**FIGURE 10:**
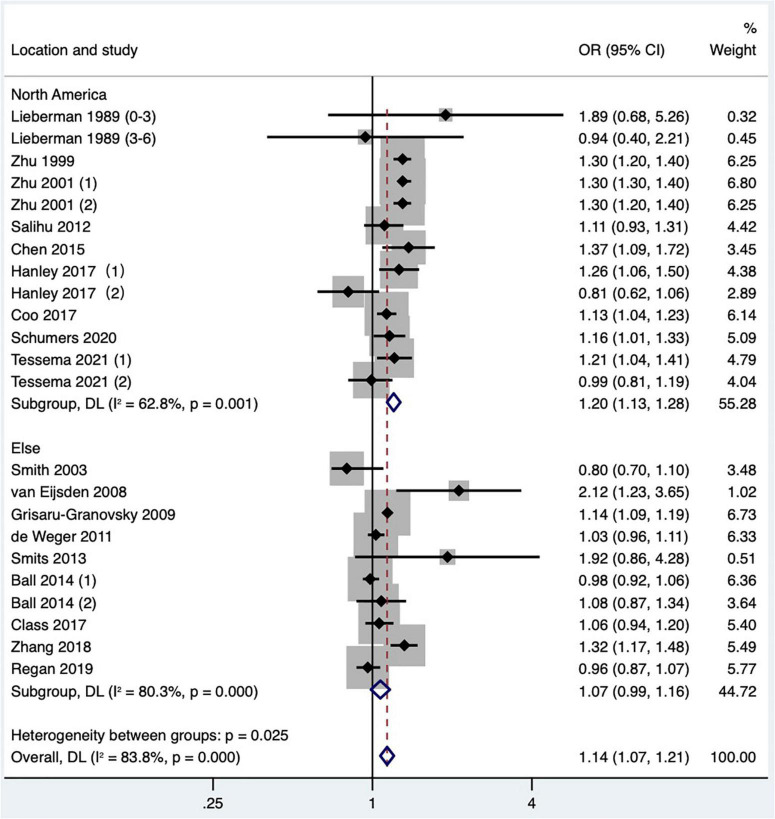
Subgroup analysis of the effect of short IPI on SGA. Lieberman et al. (13) (0–3) referred to the IPI of 0–3 months vs. 18–23 months; Lieberman et al. (13) (3–6) referred to the IPI of 3–6 months vs. 18–23 months; Zhu et al. (15) (1) was white data, Zhu et al. (15) (2) was black data; Hanley et al. (36) (1) was non-matching data, and Hanley et al. (36) (2) was within-mother matching data; Ball et al. (24) (1) was non-matching data, and Ball et al. (24) (2) was within-mother matching data; Tessema et al. (48) (1) was non-matching data, and Tessema et al. (48) (2) was within-mother matching data.

Subgroup analysis of preterm birth according to study publication time showed a pooled OR of 1.30 (95% CI: 1.22–1.39) for studies published before 2010, while a pooled OR of 1.55 (95% CI: 1.48–1.62) after 2010, and there was a statistically significant difference found in the pooled OR among subgroups (*P* < 0.001; Table 2 and Figure 9). Subgroup analysis of SGA according to study area found that short IPI (IPI < 6 months) was a risk factor for SGA in North America (pooled OR: 1.20, 95% CI: 1.13–1.28), while in other studies, short IPI (IPI < 6 months) was not a risk factor for SGA (pooled OR: 1.07, 95% CI: 0.99–1.16; Table 2 and Figure 10).

**TABLE 2:** Subgroup analysis of adverse pregnancy outcomes caused by short IPI.

Hierarchical variable	Preterm birth		Very preterm birth		Low birth weight		Small for gestational-age infants		Offspring death		NICU		Congenital abnormality		Gestational diabetes		Gestational hypertension	
									
	*n*	OR (95% CI)	*n*	OR (95% CI)	*n*	OR (95% CI)	*n*	OR (95% CI)	*n*	OR (95% CI)	*n*	OR (95% CI)	*n*	OR (95% CI)	*n*	OR (95% CI)	*n*	OR (95% CI)
Publication time																		
<2010	7	1.30 (1.22∼1.39)^abc^	2	1.56 (0.88∼2.77) ^ab^	7	1.41 (1.34∼1.49) ^ab^	8	1.22 (1.12∼1.33)^ab^	2	1.73 (1.30∼2.29)	0	∼	1	1.14 (1.04∼1.24)	0	∼	0	∼
≥2010	21	1.55 (1.48∼1.62) ^abc^	11	1.86 (1.60∼2.19) ^ab^	11	1.27 (1.12∼1.44) ^ab^	15	1.10 (1.03∼1.17)^ab^	4	1.59 (1.51∼1.68)	4	1.26 (1.01∼1.57)	3	1.08 (1.02∼1.15)	6	1.06 (0.93∼1.20)^a^	6	0.95 (0.93∼0.98)
Research site																		
North America	20	1.48 (1.41∼1.56) ^ab^	9	1.81 (1.51∼2.15)^ab^	13	1.36 (1.24∼1.48) ^ab^	13	1.20 (1.13∼1.28)^abc^	4	1.59 (1.51∼1.68)	4	1.26 (1.01∼1.57)	3	1.08 (1.02∼1.15)	4	1.13 (0.94∼1.35)^ab^	4	0.95 (0.93∼0.97)
Other areas	8	1.53 (1.34∼1.74) ^ab^	4	1.91 (1.37∼2.68)^ab^	5	1.28 (1.16∼1.40) ^ab^	10	1.07 (0.99∼1.16)^abc^	2	1.73 (1.30∼2.29)	0	∼	1	1.14 (1.04∼1.24)	2	0.96 (0.89∼1.13)	2	0.99 (0.92∼1.06)
Type of research																		
Cohort	28	1.49 (1.42∼1.57)	13	1.82 (1.55∼2.14)	18	1.33 (1.24∼1.43)	23	1.14 (1.07∼1.21)	4	1.60 (1.51∼1.69)	4	1.26 (1.01∼1.57)	3	1.09 (1.03∼1.15)	6	1.06 (0.93∼1.20)^a^	6	0.95 (0.93∼0.98)
Case–control	0	∼	0	∼	0	∼	0	∼	2	1.61 (1.11∼2.36)	0	∼	1	1.15 (1.03∼1.28)	0		0	∼
Research design																		
Non-matched	26	1.53 (1.46∼1.60)^abc^	13	1.82 (1.55∼2.14)	16	1.40 (1.33∼1.47) ^abc^	20	1.16 (1.09∼1.23)^abc^	6	1.60 (1.51∼1.69)	3	1.29 (1.01∼1.65)^ab^	4	1.10 (1.05∼1.16)	4	1.06 (0.91∼1.24)^ab^	4	0.95 (0.93∼0.98)
Matched	2	0.94 (0.75∼1.18)^abc^	0	∼	2	0.76 (0.43∼1.36) ^abc^	3	0.97 (0.84∼1.13)^c^	0	∼	1	1.11 (0.78∼1.59)	0	∼	2	1.07 (0.71∼1.63)^ab^	2	0.99 (0.89∼1.10)

^a^*I*^2^ > 50% in subgroup;

^b^*P* < 0.05 in the heterogeneity test within subgroup;

^c^*P* < 0.05 in the heterogeneity test between subgroups.

### Short interpregnancy interval on adverse maternal outcomes

#### The effect of short interpregnancy interval on gestational diabetes

**FIGURE 11:**
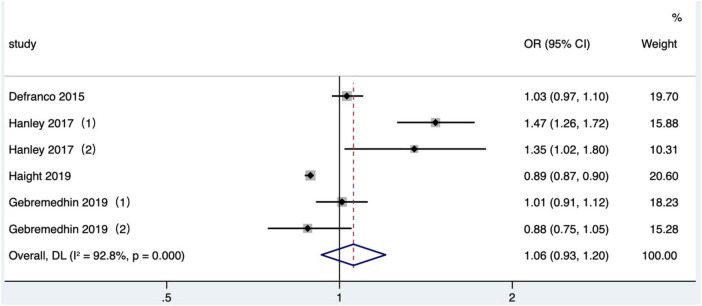
Meta-analysis forest map of the effect of short IPI on gestational diabetes. Hanley et al. (36) (1) was non-matching data, and Hanley et al. (36) (2) was within-mother matching data; Gebremedhin et al. (39) (1) was non-matching data, and Gebremedhin et al. (39) (2) was within-mother matching data.

Four articles (26, 36, 39, 41) (six research data) published after 2010 were included in the meta-analysis. Gestational diabetes was determined based on ICD-9 or ICD-10 in 2 studies (36, 39) and referred to maternal diabetes information recorded in birth certificates in the other studies (26, 41). The crude OR was calculated and included in a quantitative combination based on the numbers of maternal gestational diabetes in different IPI subgroups reported in one study (26), as the adjusted OR values were not reported. The result was demonstrated that short IPI (IPI < 6 months) was not a risk factor for gestational diabetes (pooled OR: 1.06, 95% CI: 0.93–1.20; Figure 11). According to the research site, no significant difference was found in the pooled OR between subgroups (Table 2). After removing each study one by one, the pooled OR values varied from 0.98 to 1.11, with the minimum 95% CI lower limit of 0.89 and the maximum 95% CI upper limit of 1.29. There was no significant effect on the pooled OR (pooled OR: 1.06, 95% CI: 0.91–1.24) after excluding the data of with-in mother matching of the two studies (36, 39).

#### The effect of short interpregnancy interval on gestational hypertension

**FIGURE 12:**
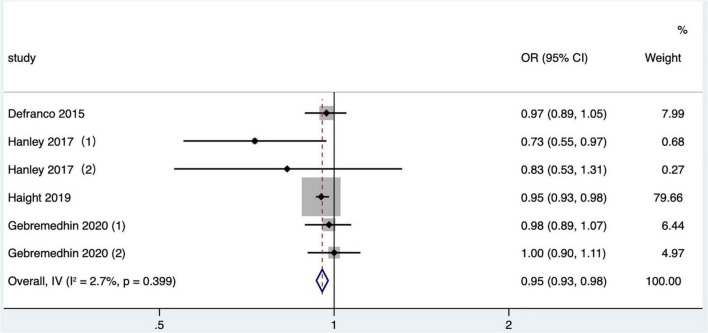
Meta-analysis forest map of the effect of short IPI on gestational hypertension. Hanley et al. (36) (1) was non-matching data, and Hanley et al. (36) (2) was within-mother matching data; Gebremedhin et al. (46) (1) was non-matching data, and Gebremedhin et al. (46) (2) was within-mother matching data.

Four articles (26, 36, 41, 46) (six research data) published after 2010 in North America were included in the meta-analysis. In two studies (26, 41), the outcome indicator was gestational hypertension based on data recorded in birth certificates, one study (36) was eclampsia or preeclampsia, and one study (46) was hypertensive disorders, which included preeclampsia and gestational hypertension without proteinuria, based on ICD-9 or ICD-10. The crude OR was calculated and included in a quantitative combination based on the numbers of maternal gestational hypertension in different IPI subgroups reported only in one study (26), as the adjusted OR values were not reported. The meta-analysis used a fixed effects model. The results manifested that short IPI (IPI < 6 months) was not a risk factor for gestational hypertension (pooled OR: 0.95, 95% CI: 0.93–0.98; Figure 12). After removing each study detail by detail, the pooled OR values varied from 0.95 to 0.97, with the minimum 95% CI lower limit of 0.92 and the maximum 95% CI upper limit of 1.02. The pooled OR value changed a little (pooled OR: 0.95, 95% CI: 0.93–0.98) after excluding the data of within-mother matching of the Hanley et al. (36) study.

#### Effect of short interpregnancy interval on uterine rupture

Two studies (17, 45) met the inclusion criteria. One study (17) was based on 17 hospitals in the United States, which limited the outcome of previous pregnancy to cesarean. The result showed that short IPI (IPI < 6 months) was associated with a higher risk of uterine rupture compared with IPI of 18–60 months (OR: 3.05, 95% CI: 1.36–6.87). De Silva and Thoma (45) found that short IPI after live birth was associated with a higher risk of uterine rupture compared with the reference IPI group (OR: 2.78, 95% CI: 2.29–3.39). Due to the obvious differences between the research populations and the reference group, the quantitative synthesis was not performed.

#### Influence of short interpregnancy interval on premature rupture of membranes

One study (31) met the inclusion criteria, but the original paper only reported the incidence of premature rupture of membranes in different IPI groups, and did not report the OR value. So it was not included in the multivariate statistical model for analysis.

#### Impact of short interpregnancy interval on maternal morbidity

Five studies (17, 37, 41, 45, 50) have reported the association between short IPI and comprehensive indicators of maternal morbidity. Stamilio et al. (17) defined the main incidence indicators as any one or more of the following symptoms: uterine rupture, bladder, ureter or intestinal injury, and uterine artery tear. Haight et al. (41) defined the indicator of maternal morbidity during delivery and hospitalization, as any combination of maternal transfusion, perineal laceration, uterine rupture, unplanned hysterectomy, and intensive care admission. The outcome indicators in the Schummers et al. (37) study were maternal mortality or severe maternal morbidity defined as mechanical ventilation, admission to an intensive care unit, organ failure, maternal transfusion, unplanned postpartum surgery, or death, which covered pregnancy up to 42 days postpartum. De Silva and Thoma (45) analyzed severe maternal morbidity, which included maternal transfusion, admission to intensive care unit, ruptured uterus, and third- or fourth-degree perineal laceration. Liu et al. (50) analyzed severe maternal morbidity with about 21 indicators of ICD-9. Due to methodological heterogeneity in the indicators’ definition of maternal morbidity in these studies, quantitative combinations were not included.

## Discussion

This study revealed that short IPI could lead to an increased risk of adverse pregnancy outcomes. Compared with the IPI of 18–23 months, the IPI less than 6 months after delivery was the risk factor of adverse perinatal outcomes in the next pregnancy and increased the risks of preterm birth, very preterm birth, low birth weight, small for gestational age infants, offspring death, congenital abnormality, and NICU. The previous meta-analysis showed that short IPI was associated with increased risk of preterm birth, extreme preterm birth, low birth weight, and small for gestational age (4, 6, 51), and short IPI after live birth was related to perinatal death (9), which was accordance with our research results. In recent years, it has been found that the mechanism of adverse pregnancy outcomes caused by short IPI after delivery could be attributed to the comprehensive influence of factors. The most common explanation was the maternal depletion hypothesis (52). During the short IPI, the essential nutrients for pregnancy may have not fully recovered to the previous prepregnancy levels, such as insufficient reserves for folic acid (18, 53–55) and iron (56), which may cause intrauterine growth retardation and fetal death. Other studies found that the incomplete healing of the uterine after cesarean section (17) and increased levels of the proteins-associated contraction (16) may cause premature delivery. The breast-feeding status after delivery may affect the mothers’ nutrients reservation, and the competition among siblings (57) may also affect fetal health. In addition, the women with short IPI didn’t have enough time to lose weight and were more likely to be obese at beginning of the subsequent pregnancy (36), which may also affect the fetal health in the subsequent pregnancy. However, due to the lack of comparative analysis on the maternal physical conditions at different period, including prepregnancy, pregnancy, postpartum, and lactation period, we need more studies to find out the biological mechanism of short IPI and adverse perinatal outcomes.

This study concluded that short IPI was a risk factor for NICU and congenital abnormality. NICU, as one of the comprehensive indicators of neonatal postpartum outcomes, might be caused by preterm birth, low birth weight, small for gestational age infants, and other causes, which reflected the severity of the impact of short IPI on the overall health status of neonates. There are few studies involving offspring death and NICU, and the measurement methods of indicators are inconsistent. However, given the very important clinical significance of offspring death and NICU, even though the strength of evidence provided by this meta-analysis was extremely low, it can still provide valuable information for clinical intervention trials. In our analysis, the included studies had given different definitions of congenital abnormality, and the categories of diseases involved were not completely consistent. As our meta-analysis gave an overview of short IPI and congenital abnormality, it may need more researches and data to find out the mechanism of congenital abnormality induced by short IPI.

This study found that short IPI was not related to the increased risk of gestational diabetes in mothers, which was different from the results of a systematic review published by Hutcheon et al. (7). The possible reason was that Hutcheon et al. included one study in high-resource settings, while this meta-analysis included more studies and had a wider coverage. This study showed that short IPI was not a risk factor for gestational hypertension and may lead to a reduced risk. Wainstock et al. believed that women who suffered from preterm birth, perinatal mortality, gestational diabetes, and other adverse outcomes rather than preeclampsia in the first pregnancy were likely to suffer from primary preeclampsia in the next pregnancy (58). There were many factors causing gestational hypertension and eclampsia/preeclampsia in pregnant women, especially the genetic susceptibility to the mother (59), as well as the first pregnancy with preeclampsia and the second pregnancy with preeclampsia relapse (60); therefore, the relationship between IPI and gestational hypertension might be influenced by the mixed adverse outcomes of the first pregnancy. However, few literature were included in the meta-analysis, and these literature did not effectively control the key factors, such as whether the previous pregnancy was accompanied by gestational diabetes (39, 41) and gestational hypertension (41). Therefore, the evidence strength of the association between short IPI and adverse maternal pregnancy outcomes in this meta-analysis was extremely low.

The advantages of this study were as follows.

The studies included in the meta-analysis were cohort studies and case–control studies, which provided valuable information for exploring the etiological relationship between IPI and pregnancy outcomes. Most of the research data came from the birth registration system, obstetric medical records, and other medical and health management data, which covered the vast majority of the target population in the research area, so the researches had high external validity.

The included studies controlled at least one confounding factor, especially maternal age, which ensures a higher quality of research. We used Egger’s test to evaluate the included studies and found no statistical risks of publication bias. It is worth mentioning that Egger’s test has high research significance for the meta-analysis with more than 10 articles included, but has low efficacy for the meta-analysis with less than 10 articles, such as our meta-analysis for infant death, NICU admission, congenital abnormality, gestational hypertension, and gestational diabetes.

The exposure factor (short IPI) defined by our meta-analysis was less than 6 months, and the reference group was 18–23 months or the wider group including 18–23 months, which ensured the comparability of the exposure factors of various studies (4, 61).

As we defined the exposure indicator as IPI, we checked the definition of the time interval between two consecutive pregnancies in the original articles and excluded the articles about birth interval. Adverse pregnancy outcomes between two live births would be omitted if the birth interval was only analyzed, including maternal death before 20 weeks of pregnancy (8) and adverse maternal and fetal situations after 20 weeks of pregnancy, such as maternal complications, late abortion (≥ 20 weeks of pregnancy, < 28 weeks of pregnancy), and stillbirth, which would lead to selection bias and measurement bias. Therefore, this study made clear that using IPI, rather than birth interval, made our research conclusion more accurate and was more conducive to the development of IPI-related consulting services for the target population in practical work.

This study also had the following limitations.

The original studies included in this analysis were at risk of bias in design. Because the majority of included studies were cohort studies, the quality of research design, implementation, data collection, and other links was highly required. The research sites were concentrated in the United States and Europe, while there were few studies in Africa and Asia. Due to the influence of regional economic development and medical level, the regions with a high medical level in which the research was conducted, there were differences in the population of the studies included in the meta-analysis. Most of the studies included in the meta-analysis were located in high-income countries, which made hard to compare the relationship between IPI and adverse pregnancy outcomes based on the different socioeconomic development levels. In addition, most research information came from birth registration system and perinatal system. The recorded information based on live births may ignore key fetal outcome events such as pregnancy termination and pregnancy loss between two live births, resulting in longer IPI recorded in the system than in the actual situation. Therefore, selection bias and misclassification bias may still exist in the original study even if pregnancy interval rather than birth interval was considered in this meta-analysis. China gradually launched the two-child policy at the end of 2015, and adverse events such as pregnancy termination and abortion may exist before the second live birth. Therefore, researchers did not include the data of people with pregnancy termination or pregnancy loss between two live births in the study of Zhang et al. (38) in the meta-analysis. This may abnegate useful information about adverse pregnancy outcomes associated with short IPI.

The studies with within-mother matched analysis were included in this meta-analysis (36, 39). That is, the associations between IPI and pregnancy outcome of the same mother were compared, and the research population was limited to women with at least three consecutive singleton births (at least two IPIs), which was different from other studies. Subgroup analysis found that in the unmatched group, the OR values of short IPI on preterm birth, low birth weight, and small for gestational age infants were 1.53 (95% CI: 1.46–1.60), 1.40 (95% CI: 1.33–1.47), and 1.16 (95% CI: 1.09–1.23), respectively, and the OR values in the matched group were 0.94 (95% CI: 0.75–1.18), 0.76 (95% CI: 0.43–1.36), and 0.97 (95% CI: 0.84–1.13), respectively. Short IPI was not a risk factor for preterm birth, low birth weight, and small for gestational age infants in the study of matched design, which suggested that within-mother matched study designs may attenuate the association of short IPI with adverse pregnancy outcomes. Since the within-mother (matched) analysis narrowed the study populations to women with three or more pregnancies, while other non-matched data focused on the women with two consecutive pregnancies, the difference in study population may not only be related to IPI but also affect the pregnancy outcomes. The selection bias and confounding bias of matched data cannot be ignored. However, the inclusion of these data in the meta-analysis did not result in a significant change in the pooled OR value. The matched design controlled the time-invariant factors such as maternal genetic characteristics and lifestyle. Therefore, we believe that the study using within-mother (matched) comparison method would not affect the support for association in this meta-analysis, and the results could provide clinical guidance for pregnant women with high parities. On the contrary, if the data of within-mother (matched) was discarded, the evidence support of the meta-analysis may be reduced.

We only conducted the meta-analysis for two maternal outcomes in our study. Because the literature available for the analysis was limited, only gestational diabetes and gestational hypertension were considered, and a quantitative combination of effect values was not conducted for adverse maternal outcomes such as obesity, dystocia, placental abruption, uterine rupture, and premature rupture of membranes in the meta-analysis, the influence of short IPI on adverse maternal outcomes could not be comprehensively analyzed.

Different studies had different definitions of pregnancy outcomes. For example, very preterm birth meant that the gestational age was less than 31 to less than 34 weeks at delivery, and there were differences in the definition of SGA. Therefore, the quality assurance of the results of meta-analysis on very premature birth and SGA was affected. In addition, maternal morbidity was mentioned as an outcome indicator in five studies with different definitions of morbidity, so a quantitative combination of effect values was not performed.

The grouping of IPI was defined in this meta-analysis when determining the inclusion criteria of literature, but different studies were inconsistent. Therefore, abandoning the studies that did not meet the inclusion criteria in the quantitative combination may lose the available research information (62–72).

This study did not conduct a meta-analysis on the related factors affecting the IPI, such as maternal age, parity, pregnancy intention, and social and economic status, which were important confounding factors of short IPI and adverse pregnancy outcomes. The association between short IPI and adverse pregnancy outcomes may be influenced by confounding factors.

In this analysis, the heterogeneity of the studies was prominent. Even with subgroup analyses based on the publication time, study site, study type, and matched data or not, the heterogeneity remained high. Heterogeneity mainly came from research methods, including data collection methods and data analysis methods. The original study we analyzed mainly obtained data through birth registration records, death records, and hospital records. As birth registration records may ignore miscarriage and stillbirth information between pregnancies, it may overestimate pregnancy interval and underestimate the incidence of adverse pregnancy outcomes. Data analysis methods, especially matching methods, as mentioned above, narrowed the research subjects and lead to low external validity of each study.

In a word, short IPI (IPI < 6 months) can lead to adverse perinatal outcomes, while it was not a risk factor for gestational diabetes and gestational hypertension. However, the evidence strength of the association between short IPI and maternal pregnancy outcomes was very low in this study. Therefore, we need more high-quality studies covering more comprehensive indicators of maternal and perinatal pregnancy outcomes, especially from the populous country in Asia (China). We suggest that future research use unified, comparable IPI groups, give full consideration to the key influencing factors such as the social demographic characteristics of pregnant women, pre-pregnancy and pregnancy behavior, and previous pregnancy outcomes, in order to adequately explore the causality short IPI and adverse pregnancy outcomes, and provide a reference for further intervention trial, which is also our next research plan to provide a relevant basis for WHO policies.

## Conclusion

Short IPI (IPI < 6 months) can lead to adverse perinatal outcomes, while it is not a risk factor for gestational diabetes and gestational hypertension. Therefore, more high-quality studies covering more comprehensive indicators of maternal and perinatal pregnancy outcomes are needed to ameliorate the pregnancy policy for women of childbearing age.

## Data availability statement

The original contributions presented in the study are included in the article/Supplementary material, further inquiries can be directed to the corresponding author/s.

## Author contributions

YW, CZ, XL, and YL designed the manuscript. YW and CZ drafted the protocol with input from YC, DT, and LY. YW, CZ, YC, DT, and LY extracted and analyzed the data. YW and CZ screened the literature. YL and XL revised and ensured the quality of the manuscript. All authors contributed to manuscript revision reviewed, read, and approved the submitted version.
