# Supplementary material for: Short interpregnancy interval can lead to adverse pregnancy outcomes: A meta-analysis
Source: Front Med (Lausanne). 2022 Nov 30;9:922053. doi: 10.3389/fmed.2022.922053 (PMC9747778; doi:10.3389/fmed.2022.922053)
Supplement: Supplementary file 1 [file Table_1.pdf]

Table S1 Basic characteristics of studies excluded from the meta-analysis

Author year	Location	Study type	Study population and period	Data source	Samples	Outcome of the last pregnancy	IPIs (months)	Main outcomes	Variables controlled	NOS score	Exclusion reasons
Brody et al. (1)	U.S.A	Prospective study	Women who had singleton live births and expected to deliver at Yale-New Haven Hospital during 1980 through 1982	Yale Pregnancy Outcome Study	1,683 women	Live birth	1-4, 5-8, ≥9 (reference group)	Low birth weight (<2,500g)	Maternal age, ethnicity, preterm delivery or low birth weight of prior newborn, and cigarette smoking in pregnancy	6	IPI groups did not meet the inclusion criteria
Klebanoff (2)	U.S.A	Cohort study	Women who registered for two consecutive pregnancies in the Collaborative Perinatal Project from 1959-1966	Multicenter study	5,938 women	Singleton infant delivery	<3 (reference group), 3-5.9, 6-8.9, 9-11.9, 12-14.9, 15-17.9, 18-20.9, 21-23.9, ≥24 months	Mean birthweight, low birthweight	Maternal age, education, socioeconomic index, smoking, and weight at the start of the first pregnancy, birthweight of the last child, and maternal race	6	IPI groups did not meet the inclusion criteria

Basso et al. (3)	Denmark	Cohort study	Women who had at least two live births in Denmark between 1980 and 1992	The Danish National Board of Health and Statistics Denmark, the Medical Birth Registry, Fertility Database	10,187 women	Live birth	0-4, >4-8, >8-12, >12-24, >24-36 (reference group), >36 months	LBW, PB	Age of the mother at the outcome birth, parity at the index child (one, two, three or more), change of social status between the two births, and social status of the mother at the birth of the index child	7	IPI groups did not meet the inclusion criteria
Shults et al. (4)	U.S.A	Cohort study	Infants born to black or white women ages 15-45 years with IPIs ≤24 months from 1988 to 1994	North Carolina birth certificate data	34,569 for preterm birth, 27,651 for SGA	Singleton birth	0-3, 4-12, and 13-24 months (reference group)	SGA, PB	Maternal age, marital status, years of completed education, smoking status during pregnancy, parity, history of SGA or preterm birth, and trimester when prenatal care began	6	IPI groups did not meet the inclusion criteria

Stephansson et al. (5)	Sweden	Cohort study	Women who delivered consecutive first and second singletons between 1983 and 1997	Swedish Medical Birth Register，linked to the Cause of Death Register	410,021 women	Singleton delivery	0-3, 4-7, 8-11, 12-35 (reference group), 36-71, ≥72 months	Stillbirth and early neonatal death	Smoking, maternal age, education, living arrangements of the infant's father, mother's country of birth, diabetes, hypertensive disease, year of second delivery, and outcome of the first pregnancy (stillbirth, early neonatal death, preterm or small for gestational age delivery)	7	IPI groups did not meet the inclusion criteria
Arafa et al. (6)	Egypt	Nested case–control study	Pregnant mothers attending 3 Maternal and Child Health centres between October 2001 to July 2002	Women who attend Maternal and Child Health (MCH) centres	1,202 women	Live birth	<12, 12–36, 37–48, 49–60 and >60 months (reference group)	Preterm delivery	Maternal age, history of miscarriage, vaginal bleeding, parity, antenatal care, previous preterm delivery, pregnancy weight, urinary tract infection and occurrence of anemia in the current pregnancy	6	IPI groups did not meet the inclusion criteria
Love et al. (7)	Scotland	Cohort study	Women who had a miscarriage in their first recorded pregnancy and subsequently became pregnant between 1981 and 2000	Database from the Information Services Division of the National Health Service	30,937 women	Miscarriage	<6, 6-12 (reference group), 12-18, 18-24, ≥24 months	Primary outcomes: miscarriage, live birth, termination, stillbirth, or ectopic pregnancy	Maternal age at first pregnancy, Carstairs social deprivation category, and year of first pregnancy	6	Study population were women with a history of miscarriage

Ratzon et al. (8)	Israel	Cohort study	Women who had a first singleton preterm delivery and a subsequent birth in Soroka University Medical Center 1988–2007	Database of Soroka University Medical Center	1,470 women	Singleton preterm delivery	≤6, >6 months (reference group)	Recurrent preterm birth	The complications of 2nd pregnancy requiring special care, miscarriages, young maternal age at 2nd delivery, gestational week at first delivery, fetal abnormality in 2nd pregnancy	6	IPI groups did not meet the inclusion criteria. Study population were women with a history of preterm birth
Simonsen et al. (9)	U.S.A	Cohort study	First three singleton live births in Utah between 1989 and 2007 and a preterm first or second birth.	Maternally-linked birth and fetal death records from the Utah Population Database	8,468 women	Singleton live births	<6, 6-12, 13-24, 25-36 (reference group), >36 months	Recurrent PTB	Maternal age, race, ethnicity, educational attainment, marital status, tobacco use, medical conditions, prepregnancy BMI, gestational weight gain, change in prepregnancy BMI between live births, inter-pregnancy interval, record of a fetal anomaly on the birth certificate, history of fetal death, and record of a father on the birth certificate	5	Study population were women with a history of preterm birth, which may cause selection bias and confounding bias

DeFranco et al. (10)	U.S.A	Cohort study	Singleton, non-anomalous live births at ≥20 weeks to multiparous mothers between 2006–2011	Vital statistics birth records	454 ,716 births	Live births	<6, 6-12, 12-18, ≥18 months (reference group)	Frequency of birth at each gestational week; preterm delivery <37 weeks; <39 and 40 weeks	Cigarette smoking, maternal age, race, and prior preterm birth	6	IPI groups did not meet the inclusion criteria
Wong et al. (11)	U.S.A	Clinical trial	Women with a human chorionic gonadotropine positive pregnancy test and whose last reproductive outcome was a loss from 2007 through 2011	Secondary analysis of women enrolled in the Effects of Aspirin in Gestation and Reproduction Trial	677 women	Pregnancy loss	0-3, >3-6 (reference group), >6-9, >9-12 , and >12 months	Live birth, pregnancy loss, types of pregnancy loss, and obstetric complications	Age, race, body mass index, eligibility criteria, gestational age of prior loss, and months tried to conceive for most recent pregnancy	Not applicable	Not cohort study. Outcome of the last pregnancy was not delivery

Mignini et al. (12)	Latin America	Cohort study	Parous women who delivered two consecutive infants between 1990-2009	The Perinatal Information System database of the Latin American Centre for Perinatology	894,476 women	Birth	3-11, 12-23 (reference group), 24-35, 36-47, 48-59, 60-71, 72-83, 84-95, 96-107, and 108-119 months	Primary outcomes：maternal and offspring mortality. Secondary outcomes: the main causes of death, postpartum haemorrhage, pre-eclampsia, eclampsia, and puerperal infection; the main causes of neonatal death, LBW, and PB	Maternal age, previous pre-eclampsia, previous eclampsia, previous caesarean section, previous early neonatal mortality, parity, diabetes, urinary infection, hypertension during first, second, and third trimester, and singleton birth	6	Pregnant women with IPIs <3 months were excluded. IPI groups did not meet the inclusion criteria

Ekin et al. (13)	Turkey	Case control study	Women who delivered two or more singletons between January 2008 and September 2014	The database of Tepecik Training and Research Hospital	2,758 patients and 379 controls	Singleton delivery	<6, 6-11, 12-17, 18-23, 24-35, and ≥36 months.	Spontaneous pre-term birth, PPROM, pre-eclampsia, gestational diabetes, anemia, abnormal placentation, congenital anomalies, SGA and post-partum hemorrhage	Maternal age at delivery of subsequent pregnancy, parity, body mass index before subsequent pregnancy (kg/m^2), type of conception, smoking habit, pregestational diabetes mellitus, fetal sex and outcome of previous pregnancy	6	Did not specify a reference pregnancy interval, nor did it compare the adverse outcomes between short pregnancy interval and reference pregnancy interval
Koullali et al. (14)	Netherlands	Cohort Study	Women with three sequential singleton pregnancies and a spontaneous preterm birth <37 weeks in the first pregnancy between 1999 and 2009	The Netherlands Perinatal Registry (PRN)	2,361 women	Spontaneous preterm birth	0-5, 6-11, 12-17, 18-23 (reference group), 24-59 and ≥60 months	Preterm birth (<37 weeks, <32 weeks), low birth weight (<2,500 g), and small-for-gestational age (<10th percentile)	Maternal age, non-White ethnicity, low socio economic status, artificial reproductive techniques, and year of birth	6	The outcome of the last pregnancy was preterm birth

Qin et al. (15)	China	Cohort Study	Singleton live births at ≥24 weeks' gestation for the second pregnancy delivered between July 1, 2015 and June 30, 2016 at hospital	Electronic medical records at hospital	3,309 second pregnancies	Live birth	7-24 (reference group), 25-48, 49-72, 73-96, and ≥97 months	PB, term LBW and SGA	Number of prenatal visits, maternal education level, previous cesarean section, current cesarean section, preexisting medical conditions, BMI at admission, and advanced maternal age	6	IPI groups did not meet the inclusion criteria
Delara et al. (16)	U.S.A	Cohort study	Women among Asian subgroups and Pacific Islanders in California with a first birth in 1999-2000 and a second birth before 2005	Hospital discharge data	189,931 women	Live birth	< 6, 6-18, >18 months (reference group)	Preterm birth	Maternal age, gestational age at delivery at first birth, maternal education, insurance status, number of prenatal care visits, month prenatal care started, mode of delivery, pregnancy-related conditions, pre-existing conditions, nativity, hospital type, number of pregnancy terminations, and rural or urban county of residence	7	IPI groups did not meet the inclusion criteria

Shree et al. (17)	U.S.A	Cohort study	Singleton gestations with delivery date available for the current and prior pregnancy between 2003-2013	Missouri birth certificate database	474,957	Live birth	≤6, 7-23, ≥24 months (reference group)	Preterm premature rupture of membranes (premature rupture of membranes between 16-36 weeks), gestational age at delivery	Maternal age, race, surrogates of socioeconomic status, BMI, tobacco use, a history of PTB	6	IPI grouping did not meet the inclusion criteria
Palmsten et al. (18)	U.S.A	Cohort Study	All live-birth deliveries to women with at least one previous pregnancy from 2000 to 2010	Linked birth certificate and assisted reproductive technology surveillance data	12,633 IVF deliveries and 1213,085 non-IVF deliveries	Live birth and non-live pregnancy outcome	<12, 12-<24 (reference group), 24-<60, and ≥60 months	PB, LBW and SGA	Birth year, state, maternal age, maternal race/ethnicity, maternal education, gravidity, diabetes or gestational diabetes, prepregnancy hypertension, and maternal smoking. IVF deliveries also adjusted for indications for IVF, number of previous assisted reproductive technology cycles, oocyte source, and number of embryos transferred	8	IPI groups did not meet the inclusion criteria

Hegelund et al. (19)	Denmark	Cohort study	Women had a live birth and at least one subsequent pregnancy during 1994 to 2010	The Danish Medical Birth Registry, other national registers administered by Statistics Denmark and linked to the birth data	328,577 women	Live birth	0-5, 6-11, 12-17, 18-23 (reference group), 24-59 and ≥60 months	Miscarriage, stillbirth, preterm delivery and small for gestational age	Maternal age, education , ethnicity, marital status and year of pregnancy outcome	5	The reported data was adjusted RD, not adjusted OR value
Regan et al. (20)	Finland , Norway , Australia	Cohort study	Consecutive singleton pregnancies in women whose most recent pregnancy had ended in stillbirth of at least 22 weeks' gestation from 1980-2015	The Medical Birth Register of Finland, the Medical Birth Registry of Norway, the Midwives Notification System in Western Australia	14,452 births	Stillbirth	<6, 6-11, 12-17, 18-23, 24-59 (reference group), ≥59 months	Stillbirth, preterm birth, and SGA birth	Maternal age, parity, decade of delivery, and gestational age of the previous pregnancy	6	Outcome of the last pregnancy was stillbirth

Lin et al. (21)	China	Cohort Study	Women who experienced the second delivery between 2014-2016	Medical records of hospital	9,552 women	Singleton live birth	<12, 12-23 (reference group), 24-59, 60-119, and ≥120 months	Gestational diabetes, premature membrane rupture, gestational hypertension, preeclampsia, placenta previa and postpartum hemorrhage. Neonatal outcome :PB, LBW, SGA, LGA, macrosomia, the Apgar score	Maternal age at the index delivery, prepregnancy BMI, educational level, conception method, birth place, previous cesarean section, and previous abortion history	7	IPI grouping did not meet the inclusion criteria

Kalengo et al. (22)	Tanzania	Cohort study	Women who delivered 2 or more consecutive singletons between 2000 and 2015	Maternally-linked data from KCMC (hospital) Medical Birth Registry	5,946 deliveries	Singleton delivery	<24, 24-59 (reference group), >59 months	Recurrence of preterm birth (two or more deliveries of live babies before 37 completed weeks of gestation)	Birth weight, pre-eclampsia, maternal age, maternal education level, residence, occupation, alcohol use in pregnancy, clinical subtypes, inter-pregnancy interval (IPI) and premature rupture of membranes in the index pregnancy	6	IPI grouping did not meet the inclusion criteria
Sanga et al. (23)	Tanzania	Cohort study	Women with at least two consecutive births who delivered at Kilimanjaro Christian Medical Centre from 2000–2015	Kilimanjaro Christian Medical Centre (KCMC) Medical Birth registry data	7,995 births from 6,612 mothers	Delivery	<24, 24-59 (reference group), >59 months	Anemia during pregnancy, post-partum hemorrhage and pre-eclampsia	Mother's age, education level, occupation, religion, marital status, tribe, current residence, pregnancy type, family planning use, alcohol use, anemia in previous pregnancy, PPH in previous pregnancy, pre-eclampsia in previous pregnancy, referral status, delivery mode, parity, any ANC visit, number of ANC visits and death of a preceding child	6	IPI grouping did not meet the inclusion criteria

Swaminathan et al. (24)	58 low-income and middle-income countries	Cohort study	Women had at least two births in the 5-year period preceding the survey (ie, July 1997 to April 2018)	Demographic and Health Surveys (2002–18) from 58 LMICs	338,223 for between-mothers, 77,600 for within-mothers	Birth	<6, 6-11, 12-17, 18-23 (reference group), and 24-59 months	Stillbirth (fetal death occurring after at least 7 months of pregnancy）	Wealth, age, region, education, outcomes of previous pregnancies, and previous inter pregnancy interval duration	6	The reported data was RD, not OR value
Gebremedhin et al. (25)	Australia	Cohort study	Mothers who had their first two consecutive births,1980-2015	The Midwives Notification System	169,896 mothers	Birth	<6, 6-11, 12-17, 18-23 (reference group), 24-59, and ≥60 months	Preeclampsia and gestational hypertension without proteinuria	Birth year, marital status, race/ethnicity, SES, known chronic diabetes, gestational diabetes, known obesity history and partner change status at recent birth	7	Duplication with the included studies. The OR value of the association between IPI groups and outcomes was not reported

References

1. Brody DJ, Bracken MB. Short interpregnancy interval: a risk factor for low birthweight. *Am J Perinatol.* (1987) 4:50-4. doi: 10.1055/s-2007-999736.

2. Klebanoff MA. Short interpregnancy interval and the risk of low birthweight. *Am J Public Health*. (1988) 78:667-70. doi: 10.2105/ajph.78.6.667.

3. Basso O, Olsen J, Knudsen LB, Christense K. Low birth weight and preterm birth after short interpregnancy intervals. *Am J Obstet Gynecol*. (1998) 178:259-63. doi: 10.1016/s0002-9378(98)80010-0.

4. Shults RA, Arndt V, Olshan AF, Martin CF, Royce RA. Effects of short interpregnancy intervals on small-for-gestational age and preterm births. *Epidemiology*. (1999) 10:250-4. doi: 10.2307/3703591

5. Stephansson O, Dickman PW, Cnattingius S. The influence of interpregnancy interval on the subsequent risk of stillbirth and early neonatal death. *Obstet Gynecol*. (2003) 102:101-8. doi: 10.1016/s0029-7844(03)00366-1

6. Arafa MA, Alkhouly A, Youssef ME. Influence of inter-pregnancy interval on preterm delivery. *Paediat Perinat Epidemiol*. (2004) 18:248-52. doi: 10.1111/j.1365-3016.2004.00564.x.

7. Love ER, Bhattacharya S, Smith NC, Bhattacharya S. Effect of interpregnancy interval on outcomes of pregnancy after miscarriage: retrospective analysis of hospital episode statistics in Scotland. *BMJ*. (2010) 341:c3967. doi: 10.1136/bmj.c3967

8. Ratzon R, Sheiner E, Shoham-Vardi I. The role of prenatal care in recurrent preterm birth. *Eur J Obstet Gynecol Reprod Biol*. (2011) 154:40-4. doi: 10.1016/j.ejogrb.2010.08.011

9. Simonsen SE, Lyon JL, Stanford JB, Porucznik CA, Esplin MS, Varner MW. Risk factors for recurrent preterm birth in multiparous Utah women: a historical cohort study. *BJOG*. (2013) 120:863-72. doi: 10.1111/1471-0528.12182

10. DeFranco EA, Ehrlich S, Muglia LJ. Influence of interpregnancy interval on birth timing. *BJOG*. (2014) 121:1633-40. doi: 10.1111/1471-0528.12891

11. Wong LF, Schliep KC, Silver RM, Mumford SL, Perkins NJ, Ye A, et al. The effect of a very short interpregnancy interval and pregnancy outcomes

following a previous pregnancy loss. *Am J Obstet Gynecol*. (2015) 212:375.e1-11. doi: 10.1016/j.ajog.2014.09.020. Epub 2014 Sep 20.

12. Mignini LE, Carroli G, Betran AP, Fescina R, Cuesta C, Campodonico L, et al. Interpregnancy interval and perinatal outcomes across Latin America from 1990 to 2009: a large multi-country study. *BJOG*. (2016) 123:730-7. doi: 10.1111/1471-0528.13625. Epub 2015 Sep 24.

13. Ekin A, Gezer C, Taner CE, Ozeren M, Mat E, Solmaz U. Impact of interpregnancy interval on the subsequent risk of adverse perinatal outcomes. *J Obstet Gynaecol Res*. (2015);41:1744-51. doi: 10.1111/jog.12783

14. Koullali B, Kamphuis EI, Hof MH, Robertson SA, Pajkrt E, de Groot CJ, et al. The effect of interpregnancy interval on the recurrence rate of spontaneous preterm birth: a retrospective cohort study. *Am J Perinatol*. (2017) 34(2):174-82. doi: 10.1055/s-0036-1584896. Epub 2016 Jul 1.

15. Qin C, Mi C, Xia A, Chen WT, Chen C, Li Y, et al. A first look at the effects of long inter-pregnancy interval and advanced maternal age on perinatal outcomes: a retrospective cohort study. *Birth*. (2017) 44:230-7. doi: 10.1111/birt.12289

16. Delara RMM, Madden E, Bryant AS. Short interpregnancy intervals and associated risk of preterm birth in Asians and Pacific Islanders. *J Matern Fetal Neonatal Med*. (2018) 31:1894-9. doi: 10.1080/14767058.2017.1331431. Epub 2017 Jun 6.

17. Shree R, Caughey AB, Chandrasekaran S. Short interpregnancy interval increases the risk of preterm premature rupture of membranes and early delivery. *J Matern Fetal Neonatal Med*. (2018) 31:3014-20. doi: 10.1080/14767058.2017.1362384. Epub 2017 Aug 9.

18. Palmsten K, Homer MV, Zhang Y, Crawford S, Kirby RS, Copeland G, et al. In vitro fertilization, interpregnancy interval, and risk of adverse perinatal outcomes. *Fertil Steril*. (2018) 109:840-848.e1. doi: 10.1016/j.fertnstert.2018.01.019

19. Hegelund ER, Urhoj SK, Andersen AN, Mortensen LH. Interpregnancy interval and risk of adverse pregnancy outcomes: a register-based study of 328,577 pregnancies in Denmark 1994-2010. *Matern Child Health J*. (2018) 22:1008-1015. doi: 10.1007/s10995-018-2480-7

20. Regan AK, Gissler M, Magnus MC, Håberg SE, Ball S, Malacova E, et al. Association between interpregnancy interval and adverse birth outcomes in

women with a previous stillbirth: an international cohort study. *Lancet*. (2019) 393:1527-35. doi: 10.1016/s0140-6736(18)32266-9

21. Lin J, Liu H, Wu DD, Hu HT, Wang HH, Zhou CL, et al. Long interpregnancy interval and adverse perinatal outcomes: a retrospective cohort study. *Sci China Life Sci*. (2020) 63:898-904. doi: 10.1007/s11427-018-9593-8. Epub 2019 Nov 5.

22. Kalengo NH, Sanga LA, Philemon RN, Obure J, Mahande MJ. Recurrence rate of preterm birth and associated factors among women who delivered at Kilimanjaro Christian Medical Centre in Northern Tanzania: a registry based cohort study. *PLoS One*. (2020) 15:e0239037. doi: 10.1371/journal.pone.0239037

23. Sanga LA, Mtuy T, Philemon RN, Mahande MJ. Inter-pregnancy interval and associated adverse maternal outcomes among women who delivered at Kilimanjaro Christian Medical Centre in Tanzania, 2000-2015. *PLoS One*. (2020) 15:e0228330. doi: 10.1371/journal.pone.0228330

24. Swaminathan A, Fell DB, Regan A, Walker M, Corsi DJ. Association between interpregnancy interval and subsequent stillbirth in 58 low-income and middle-income countries: a retrospective analysis using Demographic and Health Surveys. *Lancet Glob Health*. (2020) 8:e113-e122. doi: 10.1016/s2214-109x(19)30458-9

25. Gebremedhin AT, Tessema GA, Regan AK, Pereira G. Association between interpregnancy interval and hypertensive disorders of pregnancy: effect modification by maternal age. *Paediatr Perinat Epidemiol*. (2021) 35:415-24. doi: 10.1111/ppe.12774
