# Supplementary material for: Short interpregnancy interval can lead to adverse pregnancy outcomes: A meta-analysis
Source: Front Med (Lausanne). 2022 Nov 30;9:922053. doi: 10.3389/fmed.2022.922053 (PMC9747778; doi:10.3389/fmed.2022.922053)
Supplement: Supplementary file 2 [file Table_2.pdf]

Table S2 Basic characteristics of studies included in the meta-analysis

Author year	Location	Study type	Study population and period	Data source	Samples	Outcome of the last pregnancy	IPIs (months)	Main outcomes	Variables controlled	NOS score
Lieberman et al. (13)	U.S.A	Cohort study	Puerpera with full-term pregnancy between August 1977 and March 1980	Data based on the hospital cohort	4,489	Full-term live birth	＜3, 3-6, 6-12, 12-18, 18-24 (reference group), 24-36, 36-48, 48-60, 72-96, >96	SGA (birth weight ＜ 10th percentile for gestational age)	Maternal age, social assistance, race, educational level, smoking, alcohol consumption, pre-pregnancy BMI, weight gain during pregnancy, maternal height, chronic hypertension, and infant sex	8

Lang et al. (14)	U.S.A	Cohort study	Women whose previous pregnancy resulted in a full-term birth from August 1977 to March 1980	Medical records from Boston Hospital	4,467 women	Full-term live birth	≤3, 4-6, 7-12, 13-18,19-24, 25-36 (reference group), 37-48, ≥49 months	Preterm birth (<37 weeks' GA)	Age, insurance status, race, education, marital status, prenatal care, whether the pregnancy was planned, smoking, prepregnant weight, preexisting diabetes, uterine anomaly, parity, diethylstilbestrol exposure, cervical incompetence, previous induced abortions, urinary tract infection, maternal hematocrit and vaginal bleeding or the presence of a fetal malformation in the current pregnancy	7
Zhu et al. (2)	U.S.A	Cohort study	Women with at least one previous livebirth, who gave singleton livebirth between January 1989 and December 1996	Birth records in Utah	173,205	Live birth	0-5, 6-11, 12-17, 18-23 (reference group), 24-59, 60-100	Low birth weight (＜2,500g), premature delivery (<37 weeks' GA)) and SGA (birth weight less than 10th percentile for gestational age and sex)	Maternal age at delivery, outcome of last pregnancy, the number of live babies with livebirth deliveries, the number of dead babies with livebirth deliveries, previous spontaneous abortion or induced labor, maternal height, pre-pregnancy BMI, weight gain during pregnancy, timing of entry into prenatal care, number of prenatal care follow-up, marital status, education, race or ethnicity, place of residence (rural or urban), smoking and alcohol consumption during pregnancy	6

Zhu et al. (15)	U.S.A	Cohort study	Black and white women with at least one previous singleton livebirth, who gave singleton livebirth between January 1993 and December 1998	Singleton livebirth in Michigan	435,327 (babies)	Singleton live birth	0-5, 6-11, 12-17, 18-23 (reference group), 24-59, 60-119, ≥120	Low birth weight (＜2,500g), premature delivery (<37 weeks' GA) and SGA (birth weight less than the 10th percentile for gestational age, race, sex, and parity of infants in the U.S neonatal reference population)	Race (black/white), maternal age at delivery, marital status, education level, adequacy of prenatal care, outcome of previous pregnancy (live or stillbirth), parity, smoking and alcohol consumption during pregnancy.	4
Smith et al. (16)	Scotland	Cohort study	Women who became pregnant within 5 years of first delivery and gave birth during 1992-1998	Morbidity record in Scottish	69,055	Full-term live birth	1-5, 6-11, 12-17, 18-23 (reference group), 24-59	SGA (birth weight ＜the 5th percentile for gestational age), very preterm birth (gestational age 24-32weeks), moderate preterm birth (gestational age 33-36 weeks) and perinatal death	Socioeconomic poverty, smoking, maternal age, maternal height, marital status, previous low birth weight, previous caesarean	8

Zhu et al. (3)	U.S.A	Cohort study	Women with two consecutive singleton deliveries between January 1989 and December 2000	Michigan livebirth data	565,911	Singleton live birth	＜6, 6-11, 12-17, 18-23 (reference group), 24-59, 60-95, 96-136	Low birth weight (＜ 2,500g)	Maternal age, race, education level, prenatal care utilization, smoking and alcohol consumption during pregnancy, outcome of previous pregnancy, number of previous livebirths, father information on birth certificate (to clarify marital status and education)	7
Stamilio et al. (17)	U.S.A	Cohort study	Pregnancy women with at least one prior cesarean birth delivered by cesarean during 1995-2000	A multi-center retrospective cohort study based on medical records at 17 hospitals in the Northeastern United States	13,331	cesarean	＜6, 6-11, 12-17, 18-59, ≥60	Uterine rupture, the main maternal morbidity (any one or more of the following symptoms: uterine rupture, bladder, ureter or intestinal injury, and uterine artery tear) and maternal blood transfusion	Number of previous cesarean birth, previous vaginal deliveries, gestational age at delivery, type of hospital, anemia, smoking, maternal age, race, parity, health insurance status and type of delivery	5
van Eijsden et al. (18)	Netherlands	Cohort study	The parous women with at least one previous singleton livebirth, who gave livebirth between January 2003 and March 2004	Data from a questionnaire conducted in the community	3,153	Live birth	0-5, 6-11, 12-17, 18-23 (reference group), 24-59, ≥60	Birth weight (continuous variable), SGA (birth weight less than 10th percentiles for same gestational age, sex, and parity)	Maternal age, maternal height, BMI during pregnancy, pregnancy plan, and alcohol consumption before or during early pregnancy, smoking before or during early pregnancy, psychological pressure, cohabitation status, years of education, ethnicity/race	7

Grisaru-Granovsky et al. (19)	Israel	Cohort study	The parous women with at least one previous livebirth during 1993-2005, delivered between 2000 and 2005	Based on birth registration data from all birth centers in Israel	440,838 (livebirths), 533,206 (mothers)	Live birth	0-5, 6-11, 12-17, 18-23 (reference group), 24-59, ≥60	Preterm birth (<37 weeks' GA), very preterm birth (＜33 weeks' gestation), SGA (birth weight ＜10th centile for gestational age), very SGA (VSGA, less than 5th centile), large for gestational age (greater than 10th centile for gestational age), early neonatal death (0–6 days after delivery) and major congenital malformations	Ethnicity, maternal age at delivery, parity, previous small live births (less than 2,500g), previous large live births (lager than 3800g), maternal education level, marital status	8
de Weger et al. (20)	Netherlands	Cohort study	The parous women with one previous delivery, who gave single birth between January 2000 and December 2007	Perinatal registry system in Netherlands	263,142	Single birth	0-5, 6-11, 12-17, 18-23（reference group), ≥24	Preterm birth (<37 weeks' GA), LBW (＜2,500g in term pregnancies), and SGA (birth weight ＜10th percentile for gestational age on the basis of sex- and parity-specific Dutch standards)	Ethnicity, artificial reproductive technique, year of delivery and socioeconomic status	7

Kwon eta al. (1)	U.S.A	Case-control study	The women gave two single births during 1998-2008	Washington State birth certificates of singleton infants, hospitalization data from the state's Comprehensive Hospital Abstract Reporting System (CHARS)	10,770 (cases) 32,310 (controls)	Live birth	0-5, 6-11, 12-17, 18-23 (reference group), 24-59, ≥60	Birth defects (based on ICD-9 codes)	Maternal age at second birth, mother's parity at second birth, marital status, education level, maternal race, mother's BMI, Socioeconomic status (Kotelchuck Index), paternal age, sex of the second infant, change in paternity, characteristics of the first infant (preterm birth, low birth weight, history of a previous infant with birth defect)	7
Salihu et al. (21)	U.S.A	Cohort study	Mothers with records on consecutive singleton first and second pregnancies from 2002-2009	The Central Hillsborough Healthy Start program in Tampa, Florida linked to Florida vital statistics data	36718 mother-infant pairs（36455 for analysis）	Singleton delivery	0-5, 6-17, 18-23 (reference group), ≥24 months	LBW(＜2,500g), Preterm birth (<37 weeks' GA), SGA (birth weight <10th percentile for gestational age based on the US growth curve)	Race/ethnicity, marital status, age at first pregnancy, and educational level at first pregnancy	7

Hussaini et al. (22)	U.S.A	Case-control study	The parous women with a previous live birth, who gave singleton deliveries (cases, between January 2003 and December 2007). The parous women with a previous live birth, whose singleton infants that survived beyond age one (a random sample)	The data from the Arizona Death Certificates for infants, matching to Arizona Birth Certificates by unique birth certificate numbers that are available on each death certificate	1,466 (cases), 2,000 (controls)	Singleton live birth	＜6, 6-11, 12-17, 18-23 (reference group), 24-59, ≥60	Infant mortality	Gestational age, history of previous preterm birth, birth weight, SGA, maternal medical risks, infant sex, smoking during pregnancy, number of living children, race and ethnicity, weight gain during pregnancy, prenatal care, marital status, maternal age, insurance status, level of education and residence	6
Smits et al. (23)	Netherlands	Cohort study	The parous women with a previous singleton livebirth, delivered between January 2003 and Mach 2004	Amsterdam Born Children and their Development (ABCD)	1,659	Live birth	＜6, 6-11, 12-17, 18-23 (reference group), ≥24	Birth weight (continuous variable), SGA (birth weight below the 10th percentile for gestational age based on sex- and parity-specific standards)	Maternal age, parity, height, pre-pregnancy BMI, pregnancy intention, spontaneous pregnancy, time needed to become pregnant, date of the first prenatal care, date of birth and gestational age at birth, self-reported smoking and alcohol consumption before and/or during pregnancy, psychosocial stress, cohabitation status, education level and ethnicity	6

Ball et al. (24)	Australia	Cohort study	Mothers who delivered three liveborn singleton neonate between 1980 and 2010	The Midwives Notification System	40 441 mothers	Singleton live birth	0-5, 6-11, 12-17, 18-23 (reference group), 24-59, 60-119, and ≥120 months	Preterm birth (<37 weeks' GA), small for gestational age birth (<10th centile of birth weight by sex and gestational age), and low birth weight (<2,500g)	Maternal age, parity, and birth year. socioeconomic status, ethnicity, and the outcome of the previous birth.	7
Chen et al. (25)	Canada	Cohort study	Women who had two consecutive single births between January 1999 and December 2007	The Alberta Perinatal Health Program links to the Alberta Health and Wellness Database	46,243	Singleton live birth	0-5, 6-11, 12-17 (reference group), 18-23, 24-35, ≥36	The primary outcomes: preterm birth (<37 weeks' GA), very and extremely preterm (<34 and 28 weeks of gestation, respectively), low birth weight (<2,500g), very LBW (<1,500g), extremely LBW (<1,000g), small for gestational age and very SGA (<10th percentile for a given gestational age and less than the third percentile, respectively), perinatal death	Maternal age, smoking in pregnancy, need for social assistance, parity, presence of pre-existing or gestational diabetes, maternal and gestational hypertension, previous SGA or previous stillbirth, sex of index pregnancy, and presence of congenital anomalies in the index pregnancy	8

Defranco et al. (26)	U.S.A	Cohort study	The multiparous women with a singleton delivery between 2006 and 2011 delivered at 20-42wk' gestation	US Standard Certificate of Live Birth, 2003 Version	395,146	Singleton live birth	＜6, 6-＜12, 12-＜24 (Reference group), 24-< 60, ≥60	Primary outcome : neonatal morbidity (admission to a NICU or transfer of the neonate to a tertiary care facility). Maternal outcomes: gestational hypertension, gestational diabetes, route of delivery	Gestational age at birth, maternal race, age, and prior preterm birth	7
Nerlander et al. (27)	U.S.A	Cohort study	Women with singleton second live births aged less than 20 years, delivered during 2007-2008	The US National Center for Health Statistics	79,081	Singleton live birth	＜3, 3-5, 6-11, 12-17, 18-23 (reference group), 24-36, >36	Preterm birth (<37 weeks' GA), moderately preterm birth (32-36 weeks), very preterm birth (below 32 weeks)	Maternal age at first and second birth, maternal race/ethnicity, marital status at second birth, previous preterm birth, prenatal care utilization prior to the second birth, and smoking	7
Mburia-Mwalili et al. (28)	U.S.A	Cohort study	Live-born singleton births among Nevada resident women of child-bearing age from 2006 to 2011	Linked data from Nevada Birth Outcomes Monitoring System and birth certificate data	124,341 records	Singleton live birth	0-5, 6-11, 12-17, 18-23 (reference group), 24-35, and ≥36 month	Birth defects (ICD-9-CM, 740 to 759 range)	Age at delivery, race/ethnicity, education, sex of index infant, number of previous live births, cigarette smoking, alcohol consumption, illicit drug use, prescription drug use, and over-the-counter drug use	6

Shachar et al. (29)	U.S.A	Cohort study	Women with previous live birth or prior termination, who had singleton pregnancies during 2007-2010.	California linked birth cohort files	1,109,616	Live birth, termination	<6, 6-11, 12-17, 18-23 (reference group), 24-29, 30-35, 36-47, 48-59, ≥60	Preterm birth (20-23 weeks, 24-31 weeks and 32-36 weeks)	Maternal age (continuous), parity, race/ethnicity, prepregnancy BMI, educational attainment, medical payment, previous PTB, and smoking during pregnancy	7
Lengyel et al. (30)	U.S.A	Cohort study	Singleton live births to multiparous mothers from 2006 to 2011.	Vital statistics birth records from the Ohio Department of Health	393,441 women	Singleton live birth	<6, 6-12, 12-24 (reference group), 24-60, ≥60 months	Preterm birth (<37 weeks' GA)	Cigarette smoking, maternal age, and race	7

Apparedd y et al. (31)	U.S.A	Birth record study	Women with a previous liveborn delivery, who gave birth between January 2012 and December 2014.	The Tennessee Birth Statistical file	101,912	Live birth	＜6, 6-12, 12-18, 18-60 (reference group)	Maternal outcomes: precipitous labor, premature rupture of membranes, and cesarean section delivery. Infant outcomes: low birth weight (<2,500g), very low birth weight (<1,500g), preterm delivery (<37 weeks, <34 weeks), meconium staining, neonatal intensive care unit (NICU) admission, and death prior to 12 months of age	Maternal age, marital status, educational level, household income, race, pre-pregnancy BMI, maternal smoking, and timing of entry into prenatal care	8
McKinne y et al. (32)	U.S.A	Cohort study	Multiparous women with IPI available from January 2007 through September 2014	Linked Ohio birth and infant death records	604,217 live births to multiparous women	Birth	<6, 6-<12, 12-<24 (reference group), 24-<60, and ≥60 months	Infant mortality	Marital status, socioeconomic status (Medicaid), smoking, maternal age, and race	7

Coo et al. (33)	Canada	Cohort study	Live births and stillbirths of at least 20 weeks' gestation, and consecutive births to the same mother from April 1, 1985, to March 31, 2014	The Manitoba Population Research Data Repository at the Manitoba Centre for Health Policy houses	172 909 sibling pairs	Singleton birth	<6, 6-11, 12-17, 18-23 (reference group), 24-59, and ≥60 months	Congenital anomaly, CNS and chromosomal anomalies (ICD-9-Clinical Modification or ICD-10-Canada)	Birth year, maternal age at delivery, parity, adequacy of prenatal care, whether mother smoking, maternal prenatal alcohol consumption, maternal prenatal substance use, maternal chronic, graduated from high school, whether mother ever received income assistance, SEFI-2 score, maternal prenatal hypertension, diabetes in pregnancy, child's sex, and whether older sibling has congenital anomaly	7
Class et al. (34)	Sweden	Cohort study	The cohort consisted of secondborn and thirdborn offspring between 1973 and 2009	Swedish Medical Birth Register, Swedish population-based registries	1,050,271 (secondborn) 368,549 (thirdborn)	Singleton live birth	0-5, 6-11, 12-17, 18-23 (reference group), 24-59, ≥60	Preterm birth (<37 weeks' GA), LBW (less than 2,500g), and SGA (greater than 2 SDs below the mean weight for gestational age)	Offspring sex, year of birth, maternal and paternal age, highest education level, nationality, different father, maternal and paternal criminality, attempted suicide, substance misuse, severe mental illness, and adverse birth outcomes of the firstborn (preterm birth, LBW, and SGA)	7

Coo et al. (35)	Canada	Cohort study	Women had consecutive live births between April 1985 and March 2014	The Manitoba Population Research Data Repository	171,000	Live birth	＜6, 6-11, 12-17, 18-23 (reference group), 24-59, ≥60	The primary outcomes: preterm birth (<37 weeks' GA), low birth weight (<2,500g), and SGA (birth weight < the 10th percentile for GA and sex). The secondary outcomes: GA categories, medically indicated or spontaneous preterm birth, and GA/size for gestation categories	Birth year, child's sex, maternal age at delivery, parity, adequacy of prenatal care, education level, ever received income assistance, socioeconomic status, maternal smoking, drinking alcohol, substance abuse during pregnancy, maternal chronic hypertension, preexisting or gestational diabetes, previous pregnancy losses or stillbirths, and adverse perinatal outcomes in older siblings	8

Hanley et al. (36)	Canada	Cohort study	Women with at least three singleton deliveries (ie, two IPIs) between April 2000 and March 2015, delivered at 20-44 weeks of gestation	The British Columbia Perinatal Data Registry	38,178	Singleton birth	0-5, 6-11, 12-17, 18-23 (reference group), 24-59, ≥60	Neonatal outcomes: preterm birth (<37 weeks' GA), low birth weight (＜2,500g), and SGA (birth weight <the 10th centile for sex and gestational age), neonatal intensive care. Maternal outcomes: gestational diabetes, prepregnancy obesity, preeclampsia or eclampsia	Maternal age at each delivery, delivery year, diabetes (both pre-existing and gestational diabetes), hypertension (any diagnosis of high blood pressure), smoking during pregnancy, and history of perinatal death	6

Schummers et al. (37)	Canada	Cohort study	Women with a preinterval live birth, who had at least 2 consecutive singleton pregnancies between April 2004 and March 2014	British Columbia Perinatal Data Registry, Population Data BC	148,544	Singleton live birth	＜6, 6-11, 12-17, 18-23 (reference group), ≥24	Maternal mortality or severe morbidity through pregnancy up to 42 days postpartum; SGA birth （＜10th percentile for sex and gestational age), fetal and infant composite (stillbirth, infant death within 1 year after birth), extreme SGA birth (<3rd percentile), delivery <28 weeks), spontaneous preterm delivery at less than 37 weeks, and indicated preterm delivery at less than 37 weeks	Maternal age, nulliparity, rural residence, smoking, low neighborhood income, inadequate prenatal care, history of adverse outcomes	8

Zhang et al. (38)	China	Cohort study	Women with first (parity = 1) and second live births (parity = 2) between 26 and 43 completed weeks of gestation from January 2000 to December 2015	The Guangzhou Perinatal Health Care and Delivery Surveillance System (GPHCDSS)	227,352	Live birth	＜6, 6-＜12, 12-＜18, 18-＜24, 24-<30 (reference group), 30-<36, 36-<60, 60-<120, ≥120	PTB (gestational age <37 weeks), moderate to late preterm (32 to 36 weeks), extremely to very preterm (<32 weeks), SGA and LGA (birthweight <10th and >90th percentile, respectively, of sex- and gestational age-specific)	Maternal ethnicity, maternal age at conception of the second pregnancy, maternal education levels, calendar year of birth at second pregnancy	6
Gebremedhin et al. (39)	Australia	Cohort study	All mothers with at least three consecutive singleton births (at least two IPIs) at 20-44 weeks of gestation between January 1980 and December 2015	Midwives Notification System (MNS), Hospital Morbidity Data Collection (HMDC)	103,909 (mother), 358,046 (infants)	Singleton birth	＜6, 6-11, 12-17, 18-23 (reference group), 24-59,60-119, ≥120	Gestational diabetes	Maternal age at time of each delivery, maternal ethnicity, parity, birth year (continuous), SES (the Socio-Economic Index of Areas - Index of Relative Socio-economic Disadvantage), infant sex, marital status, history of obesity, known pre-existing hypertension and gestational hypertension	5

Gupta et al. (40)	U.S.A	Case-control study	Women with multiparous or multigravid, who had singleton pregnancies during 2006-2008 Cases: women who experienced a fetal death ≥20 completed weeks' gestation; Controls: women with a live birth ≥20 completed weeks' gestation	The Stillbirth Collaborative Research Network (SCRN)	291 (cases) 985 (controls)	Singleton pregnancy	＜6,6-11, 12-17, 18-23 (reference group), 24-59, 60-100	Stillbirth (fetal death ≥20 completed weeks' gestation)	Age, race, body mass index (BMI), education level, insurance status, smoking status, alcohol status, marital status, use of assisted reproductive technology, and prior pregnancy outcome	6

Haight et al. (41)	U.S.A	Cohort study	Women with last previous live birth, who gave singleton live births of at least 21 weeks' gestation during 2013-2016, and whose IPIs less than 24 months	The U.S. 2003 Standard Certificate of Live Birth	2,365,219	Live birth	＜6, 6-11, 12-17, 18–23 (reference group)	Preterm birth (<37 weeks' GA), gestational diabetes (diagnosis during pregnancy of glucose intolerance requiring treatment), gestational hypertension (diagnosis during pregnancy of elevation of blood pressure above normal for age, sex, and physiological condition), and maternal morbidity	Maternal age, conditions and behaviors during the IPI (such as prepregnancy smoking and body mass index), Special Supplemental Nutrition Program for Women, Infants, and Children (WIC) use and month of entry into prenatal care in the subsequent pregnancy, race and ethnicity, highest level or degree of school completed, marital status, total number of live births (including the subsequent birth), and previous preterm birth	6

Regan et al. (42)	Australia	Cohort study	Women with first and secondborn singleton live births between 1980 and 2010	The Midwives Notification System(a statutory data collection of all births in Western Australia ≥20 weeks gestation)	192,041 (sibling pairs)	Singleton live birth	＜6, 6-11, 12-17, 18-23 (reference group), 24-59, ≥60	Preterm birth (gestation <37 weeks), low birthweight (<2,500g), and small-for-gestational age (<10th percentile for birthweight by sex and gestation based on the birthweight distribution in five calendar-year blocks); preterm birth, including spontaneous and iatrogenic, moderate (gestational age of 33-36 weeks), very (gestational age of 28-32 weeks), and extreme (gestational age of <28 weeks) preterm birth	Maternal age, race, residence, socioeconomic status, and birth year	6

Lonhart et al. (43)	U.S.A	Cohort study	All singleton, live births between 20 and 41 weeks of age gestation for multiparous non-Hispanic white and black women from Birth cohort from 2007 to 2012.	California linked birth cohort files	385,919 singleton livebirths to non-Hispanic white women and 86,568 births to non-Hispanic black women	Singleton live birth	<6, 6-11, 12-17, 18-23 (reference group), 24-29, 30-35, 36-47, 48-59, and ≥60 months	Preterm birth (<37 weeks) and subtypes (20-23, 24-31 and 32-36 completed weeks)	Maternal age , parity, prepregnancy BMI, educational attainment, medical payment, initiation of prenatal care, previous PTB and smoking during pregnancy	5
Thoma et al. (44)	U.S.A	Cohort study	Non-first-born singleton births between from 2011 to 2015	Linked birth-infant death vital statistics data	9,782,029 non-first-born singleton births	Live birth	<6, 6-11, 12-17, 18-23 (reference group), 24-59, and ≥60 months	Infant death and subcategories, including early neonatal (<7 days), late neonatal (7-27 days), early postneonatal (28-90 days), early middle postneonatal (91-182 days), late middle postneonatal (183-273 days), and late postneonatal (274-365 days)	Maternal age at the current birth, race/ethnicity, level of education, maternal age at prior birth, race/ethnicity, and birth order; and, at the time of the current birth, maternal marital status, education, and county poverty level.	7

De Silva et al. (45)	U.S.A	Cohort study	Resident women with a non-first-born singleton birth from 2014 to 2017	Birth certificate data	8,599,970 women	Singleton livebirth	<6, 6-11, 12-17, 18-23 (reference group), 24-59 and ≥60 months	Severe maternal morbidity: maternal transfusion, admission to intensive care unit (ICU), ruptured uterus and third- or fourth- degree perineal laceration	Maternal age at prior birth, race/ethnicity, education level, marital status, source of payment at delivery, use of the Special Supplemental Nutrition Program for Women, Infants, and Children (WIC) during pregnancy, pre-pregnancy body mass index (BMI) and pre-pregnancy smoking. Infant characteristics included birth order, gestational age at birth, birth weight and sex	8
Gebremedhin et al. (46)	Australia	Cohort study	Women who delivered three or more consecutive singleton births between 1980 and 2015	The Midwives Notification System; the Hospital Morbidity Data Collection	103,909 mothers with 254,137 births	Birth	<6, 6-11, 12-17, 18-23 (reference group), 24-59, 60-119, ≥120 months	Preeclampsia and gestational hypertension without proteinuria	Maternal age at time of each delivery, delivery year, marital status, SES, parity, fetal sex, race/ethnicity, history of obesity, known chronic diabetes, gestational diabetes, and partner change	5

Schummers et al. (47)	Canada	Cohort study	Women with ≥2 singleton deliveries ≥20 weeks of gestational age from 1 from April 2004 to 31 March 2014	The Perinatal Data Registry and 5 other provincial databases by Population Data BC	121,151 interpregnancy intervals to 102,546 women	Live birth	<6, 6-11, 12-17,18-23 months (reference group)	Perinatal mortality (stillbirth and neonatal death within 28 days after birth), SGA (birthweight <10th percentile for gestational age and sex) , preterm delivery (<37 weeks of gestation), early preterm delivery (<28 weeks of gestation), spontaneous preterm delivery	Maternal age, low SES, BMI, and smoking in index (first) pregnancy and subsequent pregnancy	7
Tessema et al. (48)	U.S.A, Australia, Finland, Norway	Cohort study	Women who had at least two singleton births during the study during 1980-2017.	Individual-level perinatal records	5,521,206 births for the between-women analyses; 2,905,703 births for within-women analyses	Birth	0-5, 6-11, 12-17, 18-23 (reference group), 24-59, 60-119, and ≥120 months	PTB (birth <37 completed weeks of gestation), spontaneous PTB, SGA (birthweight in the lowest 10th centile based on the national birthweight distributions by gestational age and sex)	Maternal age, parity and year of birth	6

Marinovich et al. (49)	USA, Australia, Finland, Norway	Cohort study	Women with consecutive singleton live births during 1980-2017	The population-based birth and perinatal registries	3,213,855	Singleton live birth	<6, 6-11, 12-17, 18-23 (reference group), 24-59, ≥60	Preterm birth (gestation <37 weeks)	Previous term or preterm birth status, maternal age, year of birth, socioeconomic status (SES)	7
Liu et al. (50)	U.S.A	Cohort study	Sequential pregnancies to women in California during 1997 to 2012	Data from vital records linked with maternal and infant hospital discharge data from the Office of Statewide Health Planning and Development	2,203,517 for between-mother comparisons, 1,007,923 for within-mother comparisons	Delivery	<6, 6-11, 12-17, 18-23 (reference group), 24-59, and ≥60 months	Severe maternal morbidity (the Centers for Disease Control and Prevention SMM index, which comprises ICD-9, diagnosis and procedure codes corresponding to 21 indicators, using hospital discharge data ranging from the birth hospitalization to 42 days postpartum）	Infertility as reported in the index or subsequent pregnancy, and parity, gestational age, cesarean delivery, maternal age, maternal education, maternal race/ethnicity, nativity, principal source of payment for the birth, calendar year, stillbirth, SMM in the index pregnancy, Bateman score during the index pregnancy	7

Note: The references were listed in the original article.
